# Efficacy and safety of ketamine and esketamine in reducing the incidence of postpartum depression: an updated systematic review and meta-analysis

**DOI:** 10.1186/s12884-025-07186-y

**Published:** 2025-02-06

**Authors:** Moaz Yasser Darwish, Abdallah A. Helal, Yousif Ahmed Othman, Manar Alaa Mabrouk, Aya Alrawi, Taha Abd-ElSalam Ashraf, Nada K. Abdelsattar, Fatma Mohammed Sayed, Mohamed Abd-ElGawad

**Affiliations:** 1 Faculty of Medicine, Fayoum University, Fayoum, Egypt; 2 Department of Obstetrics and Gynecology, Faculty of Medicine, Fayoum University, Fayoum, Egypt

**Keywords:** Ketamine, Esketamine, Postpartum depression, PPD

## Abstract

**Background:**

Postpartum depression (PPD) is categorized by the Disorders-Fifth Edition as depression that begins during pregnancy or within the first month after giving birth. Ketamine and esketamine have shown promising results in the treatment of several depressive disorders, which suggests that they may have a role in the prevention of PPD. This systematic review and meta-analysis aim to update evidence about the efficacy and safety of using ketamine and esketamine to reduce PPD incidence.

**Methods:**

We searched four databases, PubMed, Scopus, Web of Science, and Cochrane, to collect relevant studies. We included studies which investigated the preventive effect of ketamine or esketamine on PPD among women after giving birth through caesarean or vaginal delivery. We extracted PPD occurrence rate, PPD score, pain score and side effects. Finally, a meta-analysis was conducted using RevMan software.

**Results:**

Twenty-one eligible studies were incorporated in the current systematic review and meta-analysis involving 4,389 pregnant women. Esketamine was the intervention in 14 studies, and ketamine was used in 7 studies. In subgroup analysis, both ketamine and esketamine were significantly effective in reducing the incidence of short-term PPD (ketamine: RR = 0.72, 95% CI [0.56, 0.93], *P* = 0.01; esketamine: RR = 0.43, *P* < 0.0001). Esketamine only significantly reduced the incidence of long-term PPD (RR = 0.44, *P* < 0.00001). Low doses and high doses were effective in reducing the incidence of both short-term (high dose: RR = 0.48, *P* = 0.0005; low dose: RR = 0.46, *P* = 0.002) and long-term PPD (high dose: RR = 0.54, *P* < 0.0001; low dose: RR = 0.61, *P* = 0.009). Regarding the risk of side effects, patients in the Ketamine/esketamine group showed statistically significant higher rates of developing dizziness (*P* = 0.0007), blurred vision (*P* = 0.02), vomiting (*P* = 0.004) and hallucinations (*P* = 0,002) than women in the control group.

**Conclusion:**

Both ketamine and esketamine are effective in lowering the incidence of short-term PPD. On the other hand, only esketamine is effective in reducing the incidence of long-term PPD. It is recommended to use smaller doses for a more tolerable treatment period since doses less than 0.5 mg are significantly effective. Temporary side effects such as dizziness, blurred vision, vomiting and hallucinations were reported.

**Supplementary Information:**

The online version contains supplementary material available at 10.1186/s12884-025-07186-y.

## Introduction

The postpartum period represents a time of increased vulnerability for women, necessitating prioritized healthcare support. Any unpleasant experience during this crucial period, which leads to dissatisfaction, can cause depression, affecting not only the individual but also the entire family [1]. Postpartum depression (PPD) is a prevalent complication, affecting an estimated one in seven women globally [2]. Studies suggest that over 20% of women worldwide experience PPD [3]. The typical onset of PPD occurs between six and eight weeks postpartum, potentially leading to substantial impairment of daily functioning [4].

Nowadays, PPD has emerged as a major global health concern. Despite its widespread prevalence, many women affected by this condition have not received a formal medical diagnosis [4]. While numerous factors have been linked to the development of postpartum depression, the precise aetiology of the disorder remains unclear [3].

Current PPD management primarily relies on pharmacological and psychological therapies. However, long-term medication may have adverse effects on lactating mothers, potentially affecting the neurological, emotional and behavioural development of their infants [5, 6]. Consequently, preventative strategies for PPD are of paramount importance.

The limited efficacy and delayed onset of traditional antidepressants in many individuals with PPD, coupled with potential adverse effects, underscores the critical need for novel therapeutic options to augment existing treatments. Over the past two decades, the search for more effective antidepressants has intensified, with ketamine, an N-methyl-D-aspartate (NMDA) receptor antagonist, emerging as a promising candidate [7, 8]. Notably, esketamine, an S-enantiomer of ketamine with roughly double the affinity for the NMDA receptor, is primarily used in paediatric, outpatient, and obstetric anaesthesia and perioperative pain management [9].

Ketamine has been extensively studied as a potential intervention for (PPD) due to its ability to rapidly alleviate depressive symptoms and significantly reduce the risk of suicide [10, 11].

Ketamine exhibits rapid but transient antidepressant effects, taking effect within minutes to hours of administration and peaking 24-48 h after use. Current evidence shows that even individuals who have not responded to at least two different antidepressants seem to benefit from treatment with ketamine [7, 8].

Although ketamine possesses certain advantages, its response levels and stability are yet to be reliably predicted [12, 13]. Studies have indicated that symptoms of depression can be reduced within two hours of receiving a small dose of ketamine via IV administration, with the effects lasting for two weeks [14]. Other studies suggest that co-administrating ketamine with an anaesthetic agent during caesarean delivery may prevent PPD, with its effects lasting from three days to one month [15, 16].

To date, available meta-analyses have focused exclusively on women undergoing caesarean delivery. However, it is essential to evaluate the evidence regarding the efficacy of ketamine on PPD following both caesarean and vaginal deliveries. Therefore, we performed a thorough systematic review and meta-analysis to determine the potential efficacy and safety of a sub-anaesthetic ketamine dose for preventing PPD and to investigate potential relationships between different covariates and the effect of ketamine on PPD.

## Methods

We followed the Preferred Reporting Items for Systematic Reviews and meta-analysis statements in the preparation of our review [17]. The PRISMA Checklist is presented in supplementary file 1.

### Eligibility criteria

Our systematic review included studies that met the following criteria: (1) The population studied consisted of pregnant women undergoing caesarean section or giving normal birth. (2) The study design was clinical trials or observational studies investigating the preventive effect of ketamine on postpartum depression. (3) The study reported scores of postpartum depression or the occurrence rate of postpartum depression as one of its primary or secondary outcomes. We did not apply any restrictions regarding the dose or route of administration of ketamine.

We excluded studies that did not meet the previously established inclusion criteria and those written in languages other than English, conference abstracts, and studies deemed unreliable for data extraction and meta-analysis.

### Literature search

On September 12, 2023, we systematically searched four electronic databases - PubMed, Scopus, Cochrane CENTRAL, and Web of Science. We searched over literature related to ketamine and postpartum depression, using the search strategy: (Ketamine OR S-Ketamine OR esketamine OR “2-(2-Chlorophenyl)−2-(methylamino)cyclohexanone” OR CI-581 OR “CI 581” OR CI581 OR Ketalar OR Ketaset OR Ketanest OR Calipsol OR Kalipsol OR Calypsol OR narkamon OR keta OR ketmin OR ketava OR ketalin OR ketina OR brevinaze OR keta-hameln OR Ketamines OR Spravato OR Ketalar OR Eskesia OR Ketanest-S OR Keta-S) AND (((postpartum OR Postpartum OR “Post Partum” OR Puerperium OR puerperal OR postnatal OR Post-Natal OR “Post Natal” OR “fourth trimester” OR childbirth OR delivery) AND (Depressive OR Depression OR Depressions OR Dysphoria OR “mood disorder” OR “Adjustment Disorder” OR “Affective Disorder” OR “Affective Symptoms” OR depressed)) OR (EPDS OR “edinburgh postnatal depression scale”)). The detailed search strategy is outlined in supplementary Table 1. We imposed no restrictions or filters based on publication date or study design. The literature search of the four previously mentioned electronic databases was updated on February 8, 2024.

### Study selection

Records from different databases were imported into EndNoteX9, a literature management software, in order to eliminate duplicates. Subsequently, titles and abstracts of the records are used to determine their eligibility. The full texts of the eligible records were then obtained and screened in order to select the final studies to be included. Each record was screened independently by two authors in both steps of screening. A third author resolved any disagreement.

### Data extraction

Two authors independently performed data extraction from each study of the final included studies using an online data extraction sheet including (1) general information: study ID, study design, country, time of realization, patient inclusion criteria, sample size, follow-up period after caesarean section, depression scale cut-off value, intervention and control details (2) Baseline characteristics: age, height, weight, body mass index (BMI), gestational age, duration of surgery and baseline depression score. (3) Outcomes: short-term and long-term postpartum depression score; short-term and long-term occurrence rate of postpartum depression; Day 1 and Day 2-3 pain score; adverse events including dizziness, nausea, vomiting, hallucinations, diplopia, blurred vision and headache.

We differentiated between short-term and long-term postpartum depression scores. Short-term scores were defined as those assessed up to one week after delivery, while long-term scores were those assessed four to six weeks after delivery. We used the latest score when multiple assessments were conducted within the same period. A senior author resolved any disagreement.

### Quality assessment

The quality of each study was assessed by two authors independently. We assessed the quality of the included randomized clinical trials in accordance with the newest version of the bias assessment tool in randomized controlled trials: Cochrane Risk of Bias 2 tool (RoB2) [18]. Six authors independently assessed the five domains of RoB2 in each trial: (1) randomization process. (2) deviations from intended interventions. (3) missing outcome data. (4) measurement of the outcome. (5) selection of the reported result. The authors answered each signalling question in one of the following ways: yes (Y), probably yes (PY), no (N), probably no (PN), or no information (NI). Bias in each domain were judged according to the authors’ answers to each signalling question as being one of the following: high risk, low risk, or some concerns.

On the other hand, the quality of the included non-randomized studies was assessed using the Newcastle-Ottawa Scale (NOS) for the assessment of non-randomized studies [19]. Four authors independently assessed each study regarding different domains of NOS: bias due to selection, bias due to comparability and bias due to outcome. A senior author resolved any disagreement.

### Measurement of outcome effect

Our primary outcomes were postpartum depression score and occurrence rate of postpartum depression. Researchers in all of the included studies assessed postpartum depression using the Edinburgh Postpartum Depression Scale (EPDS). Safety outcomes were the occurrence rate of nausea, vomiting, dizziness and hallucinations. Pain outcome was assessed in the included studies using the Visual Analogue Scale (VAS) or Numeric Rating Scale (NRS).

### Data synthesis

We analyzed the extracted data using RevMan software (version 5.4) for Windows. However, Open Meta-analyst software was used to perform meta-regression since heterogeneity between studies was high [20]. Change from baseline EPDS scores for both short-term and long-term PPD were calculated whenever feasible and pooled in a meta-analysis model as mean difference using the Inverse Variance method. Also, post-operative EPDS scores were pooled as mean difference and post-operative pain scores were pooled as standardized mean difference.

Short-term and long-term occurrence rates of PPD were pooled as relative risk using the Mantel–Haenszel (M–H) method. We used the fixed effect model whenever data were homogamous, but we used the random effect model whenever the assumption was that data were heterogeneous. We concluded a significant result whenever the *P* value was below 0.05 [21].

The current research did not use different estimation methods of mean and standard deviation. Whenever studies reported data unsuitable for meta-analysis or meta-regression, it was excluded from the analysis model. Studies that reported no events in both arms for a specific outcome were considered uninformative for meta-analysis.

### Assessment for heterogeneity

We assessed heterogeneity by visual inspection of the forest plot and measured it using the Chi-Square test and I-Square test. Whenever the *P* value of the Chi-square test was less than 0.1, the results were considered heterogeneous.

### Subgroup analysis

Because different studies introduced ketamine/esketamine by various doses and routes of administration in order to solve heterogeneity between the included studies, we performed a subgroup analysis based on dose and another one based on the route of administration. Additionally, a subgroup analysis based on the nature of the intervention and mode of delivery was considered whenever needed.

### Sensitivity analysis and meta-regression

In order to solve heterogeneity, we performed sensitivity analysis (leave one out) by removing a single study in each scenario. We observed the effect of excluding a single study in each scenario on the I-square test and a *P* value of the Chi-square test. We performed meta-regression in order to explore and identify sources of heterogeneity between studies. Meta-regression models were performed based on the participants’ age or the dose of ketamine applied to the intervention group.

### Publication bias

We generated a funnel plot to recognize the possibility of publication bias whenever the number of studies in a meta-analysis model allowed. The funnel plot was visually inspected in order to assess the status of publication bias.

## Results

### Data collection and study selection

**Fig. 1:**
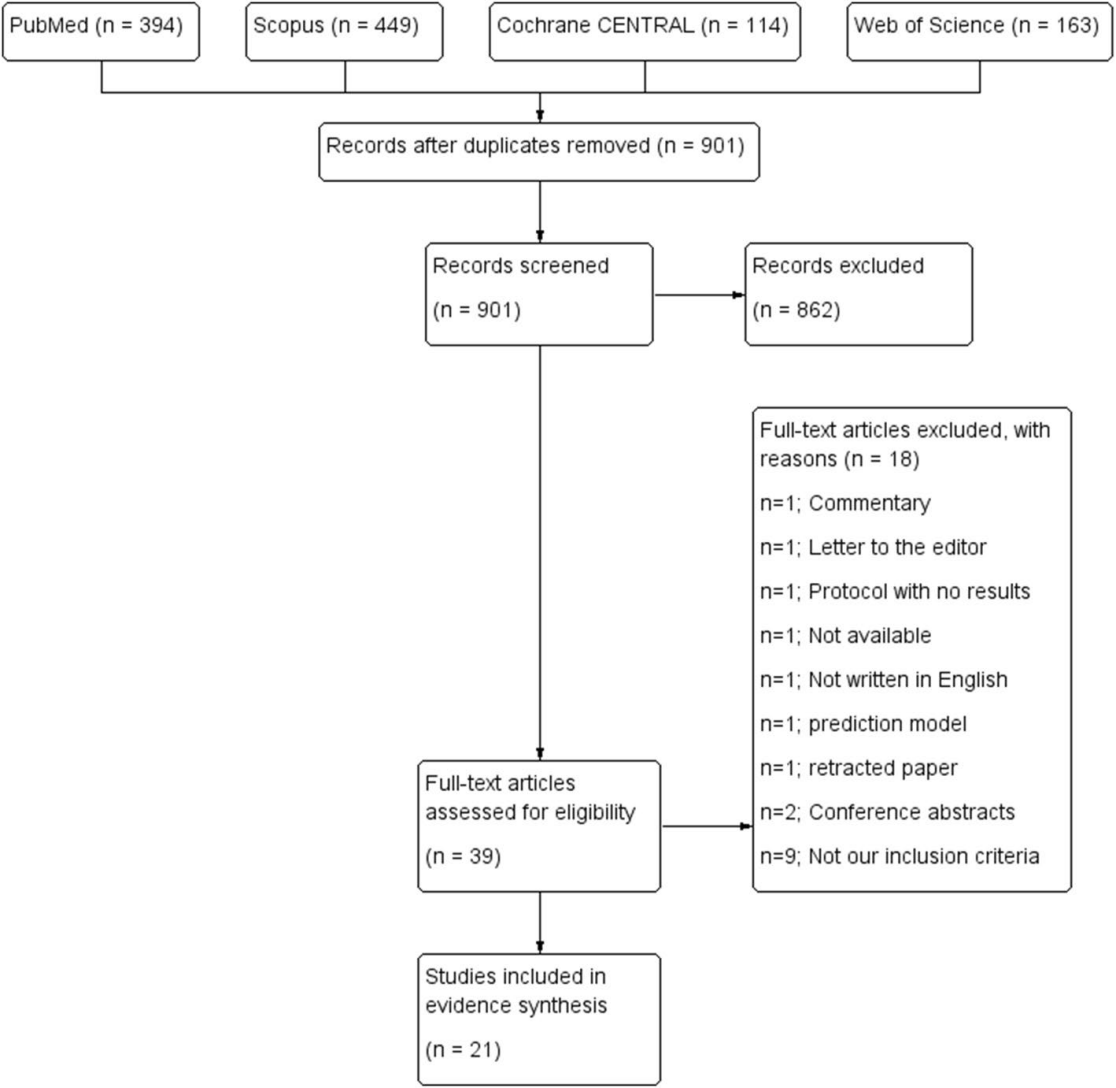
Study flow diagram

Our electronic search retrieved 1114 records. After removal of duplicates, 895 records were examined for eligibility by title and abstract screening. Only 34 records were eligible for full-text screening. Sixteen studies were eligible for evidence synthesis in that stage of our systematic review. The selection process and reasons for exclusion are demonstrated in the PRISMA flow diagram, Fig. 1. We updated our literature search on February 8, 2024, adding more 5 eligible studies. Finally, 21 eligible studies were incorporated in the current systematic review and meta-analysis.

### Characteristics of the included studies

We included a total of 18 randomised controlled trials [15, 16, 22–37] and 3 retrospective studies [38–40]. Studies were carried out between 2017 and 2024 in China (*n* = 19), Iran (*n* = 1), and the USA (*n* = 1), involving 4,389 pregnant women. Only 2 studies [25, 32] prespecified patients going through transvaginal delivery as an inclusion criterion. On the other hand, the rest of the included studies were meant to investigate the intervention in pregnant women undergoing caesarean section. Postpartum depression was assessed across all studies using the Edinburgh postnatal depression scale (EPDS) with varying follow-up points of postpartum depression across studies from 1 days to 6 months. A summary of the included studies is presented in Table 1.

**Table 1:** Summary of the general characteristics of the included studies

Study ID	Country and time of realisation	Study design	Intervention	Control	Total sample size	Anesthesia medications	Follow-up after caesarian section	Scale cut-off value	Summary of findings
Li et al. 2024 [24]	China, between January 1, 2023, to September 31, 2023.	RCT	IV infusion of Esketamine 1.5 mg/kg with 2 µg/kg sufentanil citrate is administered at a baseline rate of 2 mL/h, as well as a 1 mL on-demand bolus with a 15 min lockout interval	Anesthesia + PCIA (sufentanil citrate and tropisetron)	246	Spinal anaesthesia (ropivacaine and glucose)	6 weeks	≥ 13	On day 42, the esketamine group had an 8.2% depression rate vs. 17.6% in the control group. They also had less postoperative pain and fewer side effects.
Wang W et al. 2024 [32]	China, between October 1, 2022 and March 31, 2023	RCT	IV injection of Esketamine 0.2 mg/kg with a bolus of 10 ml, continuous infusion amount of 8 ml/h, single dose of 4 ml, locking time of 15 min and stopped after fetal disengagement	Anesthesia medications diluted to normal saline	117	Epidural anaesthesia (ropivacaine hydrochloride diluted by normal saline)	1 week and 6 weeks	> 9	The intervention group had lower pain scores and lower postpartum depression rates at 1 week and 6 weeks compared to the placebo group. No significant differences were found in side effects.
Xu et al. 2024 [34]	China, between July 1, 2020 and September 1, 2023	RCT	IV injection of Esketamine 0.2 mg/kg with 40 min continuous pumping at the beginning of the operation.	Anesthesia medications diluted to normal saline	319	Epidural anaesthesia (ropivacaine and glucose)	4 days and 6 weeks	> 9	Esketamine reduced postpartum depression at 4 days but not at 42 days. It also decreased pain and vasoactive drug use but led to a higher incidence of side effects compared to the control group.
Guo et al. 2023 [22]	China, between September 2022 and January 2023	RCT	IV infusion of Esketamine 1 mg/kg + tramadol 400 mg was diluted to 100 mL with normal saline and received at a basal rate of 2 mL/h and a 0.5mL on-demand bolus with a lockout interval of 15 min.	Butorphanol + Tramadol diluted to normal saline	170	Spinal anesthesia ( bupivacaine)	5 days and 6 weeks	≥ 10	The intervention group had lower pain scores and fewer adverse events than the control group in the first 24 h, with no difference in sleep quality, analgesic satisfaction, and EPDS scores between the groups.
Ling et al. 2023 [25]	China, between June 1, 2022, and February 28, 2023	RCT	IV injection of 0.2 mg/kg Esketamine with a bolus of 10 ml, continuous infusion amount of 8 ml/h, single dose of 4 ml, locking time of 15 min and stopped after fetal disengagement	Anesthesia medications diluted to normal saline	117	Epidural anaesthesia (ropivacaine hydrochloride diluted by normal saline)	1 week and 6 weeks		Esketamine reduced postpartum depression at one week and six weeks after delivery. There were also differences in stress and inflammation indicators at different times but similar side effects within 48 h after delivery.
Liu H et al. 2023 [38]	China, between August 2014 and June 2020	Retrospective Cohort	IV injection of ketamine 0.5 mg/kg 10 min after delivery, and the PCIA protocol was 100 µg sufentanil plus 160 mg ketamine	PCIA (sufentanil) diluted to normal saline	326		6 weeks	≥ 10	The results showed that the incidence of PPD in the two intervention groups was significantly different from that of the control group in the high-risk cohort but not in the low-risk cohort.
Liu QR et al. 2023 [26]	China, between May 2021 and December 2021	RCT	IV infusion of esketamine (0.25 mg/kg diluted to 20 mL with normal saline for the esketamine group) was administered at a rate of 40 mL/h for 30 min added to (PCIA)	PCIA (sufentanil and ondansetron) diluted to normal saline	123	Spinal anesthesia (hyperbaric bupivacaine)	3 days, 6 weeks, 3 months, and 6 months	> 12	Both groups had similar incidences of postpartum depression and anxiety risk over time. The esketamine group had lower scores for depression, pain intensity, and certain drug consumption than the control group.
Shen et al. 2023 [29]	China	RCT	IV Esketamine 0.25 mg/kg injection was given 5 min after the delivery.	Anesthesia + PCIA (Butorphanol + tramadol + Ondansetron diluted to normal saline)	202	Epidural anaesthesia (ropivacaine diluted by normal saline)	1 week, 2 weeks, and 4 weeks	≥ 9	A 0.25 mg/kg IV injection of esketamine didn’t reduce depression rates at 1, 2, or 4 weeks postpartum, but it did improve postoperative pain during exercise at 24 h.
Wang W et al. 2023 [31]	China, between April 1, 2022 and November 30, 2022	RCT	IV infusion of Esketamine 0.2 mg/kg was given 10 min after delivery of the fetus.	Anesthesia + PCIA (sufentanil + tropisetron diluted to normal saline	115	Combined spinal-epidural anaesthesia (bupivacaine and an epidural lumen tube was embedded for 4 cm.)	1 week and 6 weeks	> 9	In the intervention group, postpartum depression was significantly lower at 1 and 6 weeks after surgery—no significant difference in adverse effects at 48 h after the operation was found between the two groups.
Yang SQ et al. 2023 [36]	China, between December 2020 and January 2022	RCT	IV infusion of esketamine 2.0 mg/ kg, diluted to 100 ml, added to The PCIA At a rate of 2 ml/ h for 48 h.	Anesthesia + PCIA (sufentanil) diluted to normal saline	295	Spinal anaesthesia was administered (ropivacaine, fentanyl, and glucose)	1 week and 6 weeks	> 9	Esketamine IV infusion with PCIA reduces postpartum depression syndrome at 7 and 42 days compared to placebo. Both low- and high-dose esketamine PCIA lowers pain scores.
			IV infusion of esketamine 1.0 mg/ kg, diluted to 100 ml, added to The PCIA at a rate of 2 ml/ h for 48 h.						
Han et al. 2022 [23]	China, between September 1, 2019 and July 15, 2020	RCT	IV injection of Esketamine 0.5 mg/kg was given with a PCIA device in 100 mL.	Anesthesia + PCIA (Sufentanil + Tropisetron) + propacetamol	275	Spinal anaesthesia (ropivacaine and glucose)	3 days, 2 weeks, and 4 weeks	≥ 10	The intervention group showed lower depressive symptoms on postoperative days 3 and 14 compared to the Control group, but the difference was less noticeable by day 28. The intervention group experienced more adverse events.
Monks et al. 2022 [28]	USA, between December 2020 and August 2021	RCT	IV or SC injection of Ketamine 0.5 mg/kg after the fetus was delivered.	Anesthesia medications + SC Saline and IV Saline	23	Spinal anaesthesia (hyperbaric bupivacaine, fentanyl and preservative-free morphine.)	1 day, 2 days, 3 weeks, 6 weeks	> 12	Fewer cases of intraoperative shivering were found in the ketamine group, but there was no significant difference in the occurrence of postpartum depression screening between the groups.
Wang S et al. 2022 [30]	China, between November 23, 2017 and June 25, 2018	RCT	IV infusion of Ketamine 0.5 mg/kg was given after clamping the cord.	Anaesthesia + Postoperative epidural analgesia (ropivacaine + tramadol) + Vasopressors including ephedrine and phenylephrine + Opioids including fentanyl and sufentanil.	66	Combined spinal-epidural anaesthesia (ropivacaine)	2 days and 6 weeks	≥ 10	In this study, the ketamine group had less severe pain at 4 h postpartum and fewer instances of nausea or vomiting during surgery compared to the placebo group.
Wang W et al. 2022 [33]	China, between May 2, 2021 and December 31, 2021	RCT	IV infusion of Esketamine given in high, middle, and low doses added to (PCIA) for all women after surgery	Anesthesia + PCIA (sufentanil + totanisoltron diluted to normal saline)	156	Combined spinal-epidural anaesthesia (bupivacaine and epidural lumen tube were embedded for 4 cm.)	1 week and 6 weeks		Esketamine and sufentanil, together after a cesarean section, reduce the need for sufentanil, improve pain relief, lower the risk of postpartum depression, and do not cause more adverse effects.
Wang Y et al. 2022 [40]	China, between March 2018 and February 2020	Retrospective Study	IV infusion of Esketamine 0.35 mg/kg combined with 50 µg sufentanil citrate and 0.25 mg palonosetron hydrochloride received at a The rate was 4 mL/h, the bolus dose was 4 mL, and the lockout time was 30 min.	Anesthesia + PCIA (sufentanil citrate and palonosetron hydrochloride diluted to normal saline)	240	Spinal anaesthesia (bupivacaine hydrochloride)	1 week, 6 weeks and 3 months	> 9	The esketamine group had lower pain and EPDS scores and a lower PPD incidence than the control group. Subgroup analysis showed no significant difference between low-dose and high-dose esketamine groups.
Alipoor et al. 2021 [15]	Iran	RCT	IV injection of Ketamine 0.5 mg/kg during the induction of anaesthesia along with Nesodonal 1–2 mg/kg	Nesdonal	134		2 weeks and 4 weeks	≥ 13	EPDS scores decreased in the Ketamine-Nedonal group at four weeks post-operative and slightly increased in the Nesdonal group two weeks post-operative but significantly decreased after four weeks.
Zhang et al. 2021 [37]	China, between January 2021 and April 2021	RCT	IV injection of Esketamine 0.15 mg/kg was given 1 min before the surgery.	Anesthesia + phenylephrine + PCIA (hydromorphone diluted to normal saline)	80	Epidural anaesthesia (isobaric ropivacaine)	4 and 5days		S-ketamine lowered the ED90 of ropivacaine to 11.8 mg from 14.7 mg and reduced hypotension rates compared to ropivacaine alone.
Yao et al. 2020 [16]	China, between June 26, 2019 and July 15, 2019	RCT	IV injection of Ketamine 0.25 mg/Kg was given within 5 min after clamping the cord.	Anesthesia + IV phenylephrine + morphine diluted to normal saline	308	Spinal anaesthesia (bupivacaine in saline)	1 week, 2 weeks, and 1 month	> 9	Fewer postpartum subjects in the ketamine group had depressive symptoms at 1 week, but no differences at 2 weeks and 1 month. The ketamine group had lower pain scores at 2 days but more side effects.
Ma et al. 2019 [27]	China, between August 2014 and December 2016.	RCT	Epidural ketamine (0.5 mg/kg, diluted to 10 mL with 0.9% saline) was given (1) At 10 min of delivery and (2) After the operation (PCIA) device in a total volume of 100 mL.	Anesthesia + PCIA (Palonosetron hydrochloride + sufentanil)	654	Spinal anesthesia (ropivacaine + fentanyl + glucose)	4 days and 6 weeks	> 9	Ketamine reduced postpartum blues and depression compared to a control group. It also helped protect against depression with risk factors, including stress during pregnancy and antenatal depressive symptoms.
Xu et al. 2017 [35]	China, between October 8, 2015 and March 10, 2016	RCT	IV injection of Ketamine 0.25 mg/Kg was given within 5 min after clamping the cord.	Anesthesia + IV phenylephrine + PCIA (sufentanil diluted to normal saline)	325	Spinal anesthesia (ropivacaine plus morphine)	3days and 6 weeks	≥ 10	Intra-operative low-dose ketamine did not prevent postpartum depression, but it did significantly reduce pain scores at 6 weeks postpartum compared with a saline group.

Patients were allocated to either the ketamine/esketamine group or the control group in the included studies. Regarding the nature of the intervention, fourteen studies [22–26, 29, 31–34, 36, 37, 39, 40] used esketamine as the drug of choice in the intervention group, and seven studies [15, 16, 27, 28, 30, 35, 38] Used ketamine instead. Varying doses of ketamine/esketamine were adopted in different studies, ranging from 0.15 µg/kg to 2 mg/kg. Six studies administrated ketamine/esketamine in the form of patient-controlled intravenous analgesia (PCIA); of them, a single study added the epidural route to the PCIA. The epidural route without PCIA was considered as a route of administration by Wang W et al., 2024. The subcutaneous route was adopted only by Monks et al., 2022. The remaining thirteen studies used intravenous injection or infusion as the single route of administration of the desired intervention. The baseline characteristics of the population are provided in Table 2.

**Table 2:** Baseline characteristics of included studies

Study ID	Groups		Age (yrs)	Baseline depression score	BMI (kg/m2)	Gestational age (weeks)	Weight(kg)	Height(cm)
Li et al. 2024 [24]	Esketamine 1.5 mg/kg		28±3.1	3.6±3	**_**	**_**	**_**	**_**
	Control		27.7±4.8	4±2.1	**_**	**_**	**_**	**_**
Wang W et al. 2024 [32]	Esketamine 0.2 mg/kg		27.6±4.3	**_**	28.2±4.7	38.5±2.6	**_**	**_**
	Control		28.1±3.9	**_**	27.9±5.1	38.8±3.3	**_**	**_**
Xu et al. 2024 [34]	Esketamine 0.2 mg/kg		30.3±3.8	**_**	28.3±4.1	**_**	73.6±11.1	161.4±4.4
	Control		30.9±3.8	**_**	28.5±3.9	**_**	74.6±11.5	161.4±4.4
Guo et al. 2023 [22]	Esketamine 1 mg/kg		28[7]	2±1.1	29[4.9]	**_**	74.9±9.3	161[5]
	Control		30[9]	2.2±1.3	28.4[6.0]	**_**	75.3±10.4	161[6]
Ling et al. 2023 [25]	Esketamine 0.2 mg/kg		28.2±4.8	_	28.2±4.1	38.8±3.7	67.8±7.1	159.2±6.7
	Control		27.8±4.4	_	27.8±3.6	39±2.6	66.9±5.9	157.8±6.2
Liu H et al. 2023 [38]	Low risk	IV ketamine 0.5 mg/kg	30[28.00, 34.00]	5[3.00, 7.00]	_	_	_	158.5[155.00, 162.00]
		Control	30.5[28.00, 34.00]	5.5[4.00, 7.00]	_	_	_	159[155.00, 162.25]
	High risk	IV ketamine 0.5 mg/kg	31[28.00, 34.00]	11[10.00, 13.00]	_	_	**_**	160[156.50, 162.00]
		Control	32[28.00, 35.00]	11[9.00, 12.50]	_	_	**_**	159[155.50, 162.50]
Liu QR et al. 2023 [26]	IV ketamine 0.25 mg/kg		30.3±4.1	7(4-10)	28.4±3.7	_		_
	Control		29.8±4.2	6(3-9)	28.7±3.8	_	_	_
Lou et al. 2023 [39]	IV ketamine	overall	_	6.72±2.26	_	_	_	_
		15 μg/kg	_	6.82±2.26	_	_	_	_
		30 μg/kg	_	6.66±2.23	_	_	_	_
		45 μg/kg	_	6.68±2.25	_	_	_	_
	Control		_	6.53±2.34	_	_	_	_
Shen et al. 2023 [29]	IV Ketamine 0.25 mg/kg		28.9±3.9	6.8(0-16)	_	39.1(37-40.86)	69.4±9.6	160.7±4.5
	Control		29.6±3.9	7.34(0-19.1)	_	39(37.29-41)	68.9±8.8	159.7±3.8
Wang W et al. 2023 [31]	IV Ketamine 0.2 mg/kg		28.3±4.9	_	26.9±4.4	38.7±3.6	65.8±5.9	159.1±7.1
	Control		27.9±4.1	_	27.6±3.7	39.1±2.3	66.5±5.8	157.6±6.5
Yang SQ et al. 2023 [36]	IV esketimne	2 mg/kg	31.9±3.9	11(10.0-13.0)	26.4±2.3	_	_	_
		1 mg/kg	31.7±3.8	12(10.0- 13.0)	27.2±2.4	_	_	_
	Control		32.2±4.2	123(11.0-13.0)	27.9±2.9	_	_	_
Han et al. 2022 [23]	IV Ketamine 0.5 mg/kg		31.64±3.93	6.72±2.25	27.08±2.95	_	_	_
	Control		31.85±4.16	6.54±2.35	26.89±2.58	_	_	_
Monks et al. 2022 [28]	IV Ketamine 0.5 mg/kg		30.1±4.09	5±4.78	36±6.06	38± 1.11	94.4±12.4	162±5
	SC Ketamine 0.5 mg/kg		32.6±0.95	6.25±6.27	41.1±12	38.7± 1.11	111.1±34.9	164±5
	Control		33±6.53	8.29±4.72	32.8±7.84	37.5± 0.76	86.4±19	163±3
Wang S et al. 2022 [30]	IV ketamine 0.5 mg/kg		33±4	12[10-13]	27.4±4.1	38.7[37.9-39.4]	_	_
	Control		35±5	11[10-12]	27.5±3	39[37.9-39.7]	_	_
Wang W et al. 2022 [33]	IV ketamine 0.4 mg/kg		27.9±6.1	_	27.6±5.7	39.1[38.1-40.6]	66.5±7.8	157.6±6.5
	IV Ketamine 0.2 mg/kg		28.3±5.9	_	26.9±5.2	39.3[38.4-41.2]	65.8±8.2	159.1±7.1
	IV Ketamine 0.1 mg/kg		28.8±6.4	_	26.9±5.4	39.5[38.5-40.9]	68.3±7.2	156.7±8.2
	Control		29.1±5.5	_	27.1±6.1	39.4[38.3-41.2]	67.3±6.9	156.8±7.8
Wang Y et al. 2022 [40]	Esketamine 0.35 mg/kg		29.5±4	_	_	_	_	_
	Control		29.6±4.6	_	_	_	_	_
Alipoor et al. 2021 [15]	IV ketamine 0.5 mg/kg		27.4±4.09	13.78±3.87	_	_	_	_
	Control		28.24±4.81	13.79±4.78	_	_	_	_
Zhang et al. 2021 [37]	IV Ketamine 0.15mg/kg		32.8±5	2.9±2.3	_	39.1±0.9	69.7±7.5	162.8±4.9
	Control		31.6±3.3	3.5±2.5	_	38.9±1.1	71.4±8.9	161±4.2
Yao et al. 2020 [16]	IV ketamine 0.25 mg/ kg		30±4	_	29±3	38.57±1.29	_	_
	Control		30±3	_	28±3	38.43±1.57	_	_
Ma et al. 2019 [27]	IV ketamine 0.5 mg/kg			_	27.5±3.1	_	_	_
	Control			_	29.4±26.6	_	_	_
Xu et al. 2017 [35]	IV ketamine 0.25 mg/ kg		31±4	_	27±3	39±1.57	_	_
	Control		32±4	_	28±3	38.86±1.29	_	_

Publication bias was noted in funnel plots of both short-term and long-term occurrence of depression, evidenced by their asymmetric pattern. The asymmetric appearance was also evident in the funnel plots of long-term EPDS and adverse event dizziness, indicating the potential presence of unrepresented studies. Funnel plots are demonstrated in supplementary Fig. (1 A-1D).

### Quality assessment

#### Quality assessment of randomised controlled trials

**Fig. 2:**
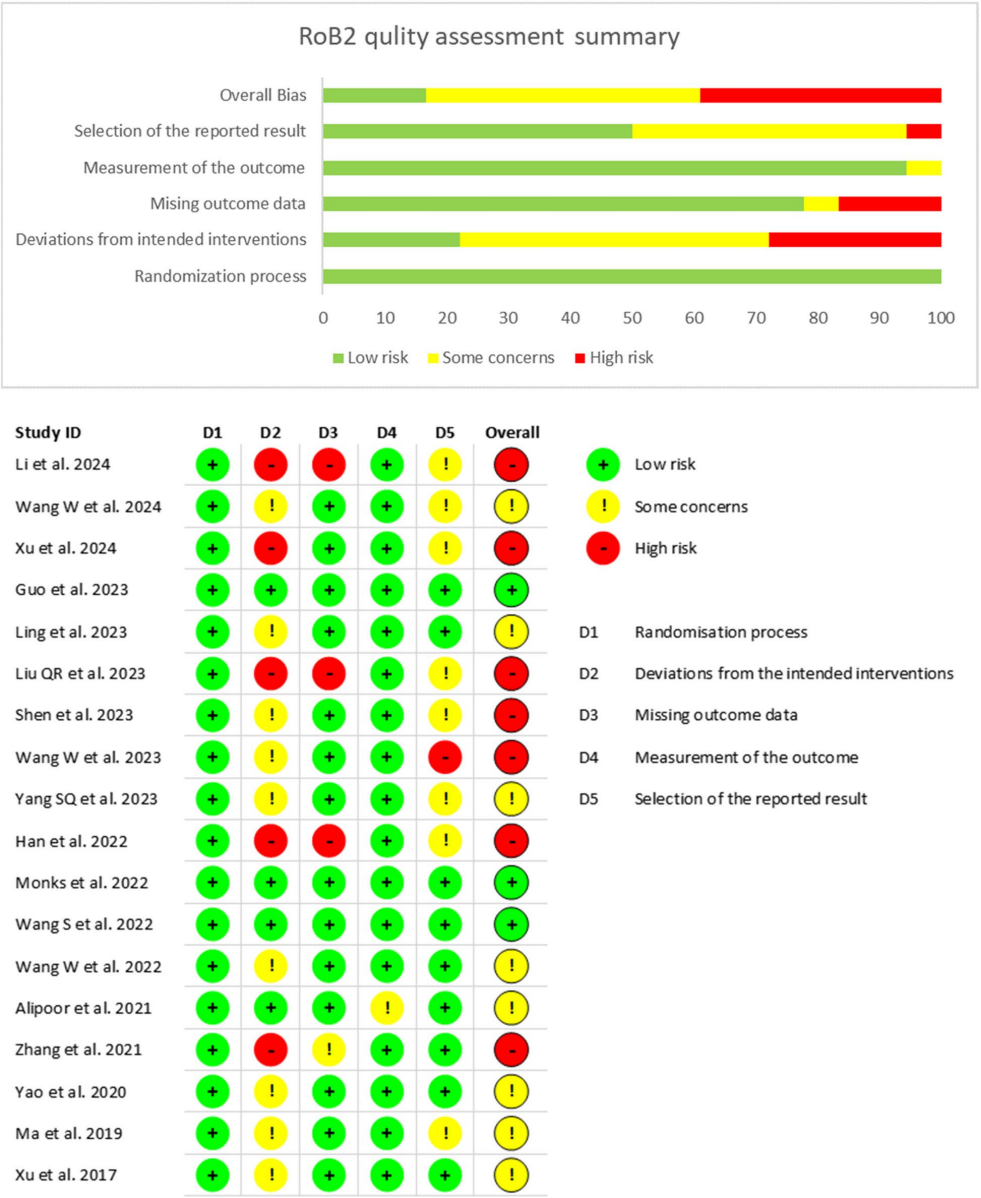
**A** risk of bias summary, (**B)** risk of bias graph

We used the ROB 2 tool to evaluate the quality of the randomised controlled trials. Figure 2A and B summarise the quality assessment and risk of bias graph, respectively. Regarding the randomisation process, we judged all of the included studies as low risk of bias.

In terms of deviations from the intended outcomes, many studies raised some concerns or were judged as high risk due to the lack of intention-to-treat analysis despite losing patients during the follow-up period. However, the loss did not exceed 5% of the total population in most of these studies, yielded them raising some concerns in this domain [16, 25, 27, 29, 31–33, 35, 36]. Five studies [23, 24, 26, 34, 37] They were judged as high risk because the loss of follow-up exceeded 5%.

Regarding missing outcome data, data were available for nearly all patients in most studies. Despite the loss of patients during follow-up and lack of intention-to-treat analysis in many studies, loss of follow-up did not exceed 5% of the population in most of them. Hence, they were judged as low risk. Li et al. 2024 [24] Liu QR et al. 2023 [26] and Han et al. 2022 [23] They were judged as high risk because data were analysed as treated despite the presence of 8%, 18% and 13% loss of the population during follow-up, respectively, with no convincing reasons regarding most of them. Zhang et al., 2021 [37]. raised some concerns due to the presence of more than 5% loss, but with accepted reasons.

Measurement of the outcome domain was judged as low risk in all of the included studies except Alipoor et al. 2021 [15]. Alipoor et al. raised some concerns due to the absence of information on whether the outcome assessors were blinded or not. However, there is no evidence to suggest that it impacted the outcome.

In terms of the selection of the reported results, many studies were judged as low risk due to adherence to a prespecified protocol and analysis plan. However, eight studies [23, 24, 26, 27, 29, 32, 34, 36] raised some concerns due to a lack of information regarding the analysis plan. Wang W et al., 2023 [31]. was judged to have a high risk of bias as a result of not adhering to their protocol in some outcomes.

#### Quality assessment of non-randomized controlled studies

The three included retrospective studies [38–40] were appraised using the Newcastle-Ottawa Scale (NOS) for non-randomized controlled studies. All of the three studies were considered of good quality. A summary of NOS scores is illustrated in supplementary Table 3.

### Outcomes

#### Occurrence of PPD

**Fig. 3:**
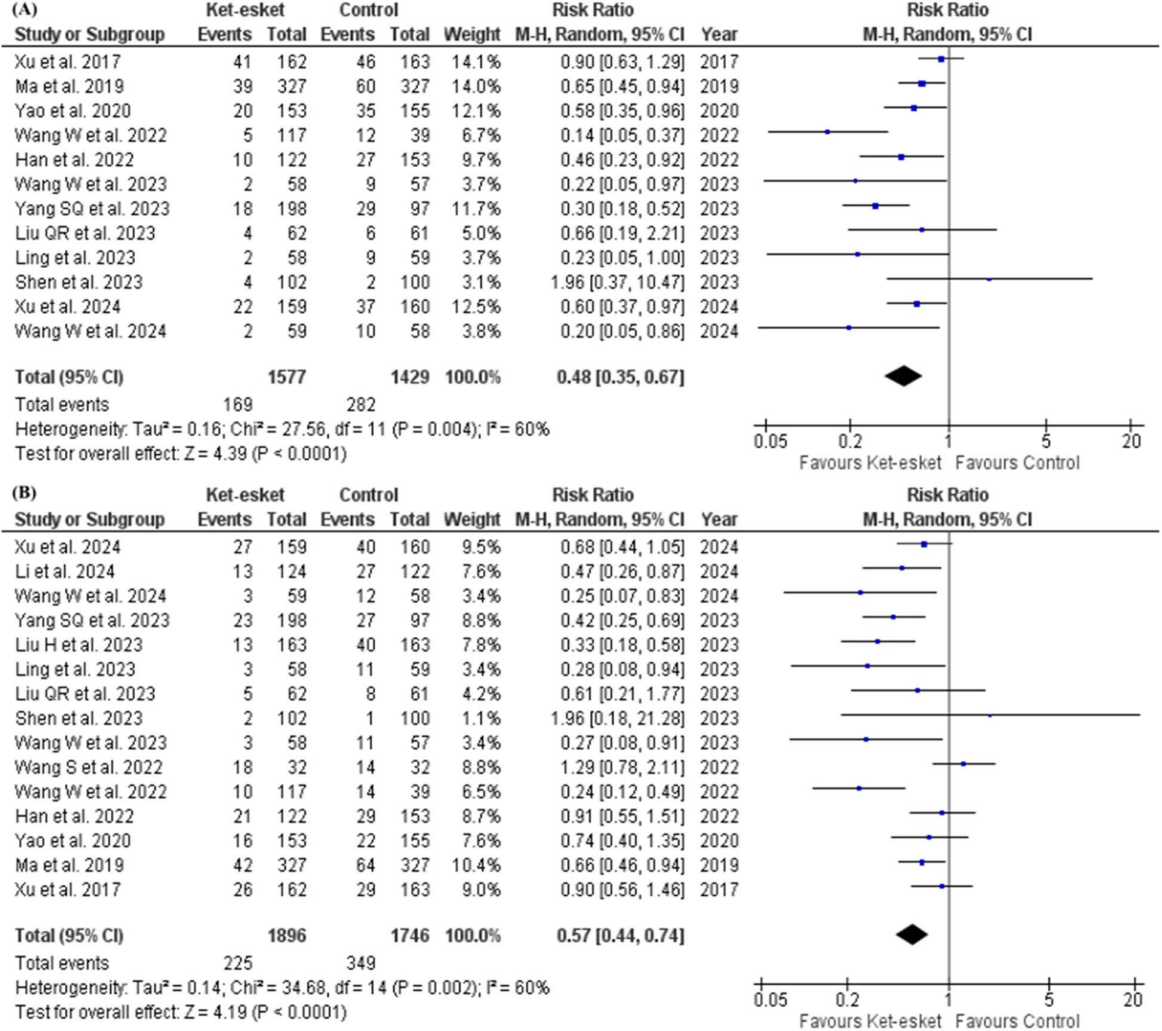
Forest plot of (**A**) short-term occurrence of PPD, (**B**)long-term occurrence of PPD

Pooled analysis of 12 studies reporting short-term PPD [16, 23, 25–27, 29, 31–36] and 15 studies reporting long-term PPD [16, 23–27, 29, 31–36, 38] demonstrated a significant reduction in both short-term and long-term PPD incidence in the ketamine-esketamine (Ket-esket) group compared to the control group (short-term PPD: RR = 0.48, 95% CI [0.35, 0.67], *P* < 0.0001; long-term PPD: RR = 0.57, 95% CI [0.44, 0.74], *P* < 0.0001). Pooled studies were not homogenous in either of the meta-analysis models (short-term PPD: I² =60%, *P* = 0.004; long-term PPD I² =60%, *P* = 0.002) (Fig. 3A and B) (Table 3). Heterogeneity was not resolved in either of the two analyses by excluding any study from the meta-analysis model.

**Table 3:** Meta-analysis summary

Outcome	Meta-analysis model	Subgrouping covariate	Analysis of subgroup	Number of studies	Estimate	95% CI	*P* value	Heterogeneity *P* value	Heterogeneity is best resolved when removed
Short-term occurrence of PPD	Double-arm meta-analysis, random effect model	Intervention dose	0.5 mg/kg or more	4	0.48	(0.32, 0.73)	0.0005	0.14	Heterogeneity remained high after removing a single study from the meta-analysis model in multiple scenarios.
			Less than 0.5 mg/kg	8	0.46	(0.28, 0.76)	0.002	0.004	
		Intervention route of administration	Intravenous	6	0.52	(0.30, 0.90)	0.02	0.06	
			PCIA	4	0.47	(0.21, 1.02)	0.06	0.003	
			Epidural	1	0.22	(0.05, 0.97)	0.05	-	
			Epidural + PCIA	1	0.65	(0.45, 0.94)	0.02	-	
		Mode of delivery	Cesarean section	10	0.52	(0.37, 0.72)	< 0.0001	0.004	
			Transvaginal delivery	2	0.21	(0.07, 0.60)	0.004	0.9	
		Nature of intervention	Ketamine	3	0.72	(0.56, 0.93)	0.01	0.29	
			Esketamine	9	0.38	(0.25, 0.57)	< 0.00001	0.07	
		-	Total	12	0.48	(0.35, 0.67)	< 0.0001	0.004	
			Sensitivity analysis	-	-	-	-	-	
Long-term occurrence of PPD	Double-arm meta-analysis, random effect model	Intervention dose	0.5 mg/kg or more	7	0.62	(0.44, 0.88)	0.008	0.004	Heterogeneity remained high after removing a single study from the meta-analysis model in multiple scenarios.
			Less than 0.5 mg/kg	8	0.51	(0.33, 0.78)	0.002	0.03	
		Intervention route of administration	Intravenous	7	0.75	(0.53, 1.08)	0.13	0.08	
			PCIA	6	0.46	(0.32, 0.67)	< 0.0001	0.05	
			Epidural	1	0.25	(0.07, 0.83)	0.02	-	
			Epidural + PCIA	1	0.66	(0.46, 0.94)	0.02	-	
		Mode of delivery	Cesarean section	13	0.6	(0.46, 0.79)	0.0002	0.002	
			Transvaginal delivery	2	0.26	(0.11, 0.62)	0.002	0.89	
		Nature of intervention	Ketamine	5	0.72	(0.48, 1.09)	0.12	0.008	
			Esketamine	10	0.48	(0.35, 0.67)	< 0.0001	0.06	
		-	Total	15	0.57	(0.44, 0.74)	< 0.0001	0.002	
			Sensitivity analysis	-	-	-	-	-	
Short-term EPDS (up to one week after delivery)	Double-arm meta-analysis of PPD score after delivery, random effect model	Intervention dose	0.5 mg/kg or more	4	−1.44	(−2.26, −0.62)	0.0005	0.001	Heterogeneity remained high after removing a single study from the meta-analysis model in multiple scenarios.
			Less than 0.5 mg/kg	5	−0.71	(−1.05, −0.37)	0.0005	0.22	
		Intervention route of administration	Intravenous	5	−0.97	(−1.61, −0.32)	0.003	0.02	
			PCIA	3	−0.96	(−1.50, −0.42)	0.0005	0.04	
			Subcutaneous	1	−4.5	(−6.41, −2.59)	< 0.00001	-	
			Epidural + PCIA	1	−0.9	(−1.55, −0.25)	0.006	-	
		Nature of intervention	Ketamine	4	−1.04	(−1.93, −0.16)	0.02	0.002	
			Esketamine	5	−0.99	(−1.39, −0.59)	< 0.00001	0.07	
		-	Total	9	−0.98	(−1.36, −0.59)	< 0.00001	0.002	
			Sensitivity analysis	-	-	-	-	-	
	Double-arm meta-analysis of change from baseline in PPD score	Nature of intervention	Ketamine	1	1.38	(−1.13, 3.88)	0.28	-	Heterogeneity remained high after removing a single study from the meta-analysis model in multiple scenarios.
			Esketamine	5	−1.45	(−2.36, −0.54)	0.01	0.0002	
		-	Total	6	−1.21	(−2.13, −0.29)	0.01	0.0005	
			Sensitivity analysis	-	-	-	-	-	
Long-term EPDS (four to six weeks after delivery)	Double-arm meta-analysis, random effect model	Intervention dose	0.5 mg/kg or more	6	−1.6	(−2.66, −0.54)	0.003	< 0.00001	Heterogeneity remained high after removing a single study from the meta-analysis model in multiple scenarios.
			Less than 0.5 mg/kg	4	−0.55	(−1.02, −0.08)	0.02	0.06	
		Intervention route of administration	Intravenous	5	−1.38	(−2.51, −0.24)	0.02	< 0.00001	
			PCIA	4	−0.9	(−1.74, −0.06)	0.04	< 0.00001	
			Subcutaneous	1	−6.39	(−8.91, −3.87)	< 0.00001	-	
			Epidural + PCIA	1	−0.65	(−1.36, 0.06)	0.07	-	
		Nature of intervention	Ketamine	5	−1.45	(−2.62, −0.28)	0.02	< 0.00001	
			Esketamine	5	−0.87	(−1.58, −0.17)	0.02	< 0.00001	
		-	Total	10	−1.04	(−1.62, −0.46)	0.0005	< 0.00001	
			Sensitivity analysis	-	-	-	-	-	
	Double-arm meta-analysis of change from baseline in PPD score	Nature of intervention	Ketamine	2	−1.85	(−3.44, −0.25)	0.02	0.4	Guo et al. 2023 [22]
			Esketamine	5	−0.75	(−1.39, −0.10)	0.02	0.01	
		-	Total	7	−0.86	(−1.47, −0.26)	0.005	0.02	
			Sensitivity analysis	6	−1.07	(−1.53, −0.61)	< 0.00001	0.31	
Day 1 pain score	Double-arm meta-analysis, random effect model	Intervention dose	0.5 mg/kg or more	4	−0.38	(−0.60, −0.17)	0.0003	0.16	Heterogeneity remained high after removing a single study from the meta-analysis model in multiple scenarios.
			Less than 0.5 mg/kg	5	−1.9	(−2.96, −0.84)	0.0005	< 0.00001	
		Intervention route of administration	Intravenous	4	−2.89	(−5.23, −0.54)	0.02	< 0.00001	
			PCIA	4	−0.44	(−0.63, −0.24)	< 0.0001	0.08	
			Subcutaneous	1	1.4	(0.23, 2.57)	0.02	-	
			Epidural	1	−1.12	(−1.51, −0.73)	< 0.00001	-	
		Mode of delivery	Cesarean section	8	−1.16	(−1.74, −0.58)	< 0.0001	< 0.00001	
			Transvaginal delivery	1	−1.12	(−1.51, −0.73)	< 0.00001	-	
		Nature of intervention	Ketamine	1	−0.36	(−1.25, 0.54)	0.43	-	
			Esketamine	8	−1.23	(−1.80, −0.67)	< 0.0001	< 0.00001	
		-	Total	9	−1.15	(−1.69, −0.62)	< 0.0001	< 0.00001	
			Sensitivity analysis	-	-	-	-	-	
Day 2–3 pain score	Double-arm meta-analysis, random effect model	Intervention dose	0.5 mg/kg or more	4	−0.5	(−0.91, −0.09)	0.02	0.0005	Heterogeneity remained high after removing a single study from the meta-analysis model in multiple scenarios.
			Less than 0.5 mg/kg	5	−2.07	(−3.08, −1.06)	< 0.0001	< 0.00001	
		Intervention route of administration	Intravenous	5	−2.73	(−4.05, −1.41)	< 0.0001	< 0.00001	
			PCIA	4	−0.42	(−0.71, −0.14)	0.004	0.003	
			Subcutaneous	1	−0.96	(−2.05, 0.13)	0.09	-	
		Nature of intervention	Ketamine	2	−1.24	(−1.47, −1.00)	< 0.00001	0.53	
			Esketamine	7	−1.31	(−1.87, −0.66)	< 0.0001	< 0.00001	
		-	Total	9	−1.31	(−1.87, −0.75)	< 0.00001	< 0.00001	
			Sensitivity analysis	-	-	-	-	-	
Nausea	Double-arm meta-analysis, random effect model	-	Total	6	0.83	(0.32, 2.16)	0.7	0.02	Zhang et al. 2021 [37]
			Sensitivity analysis	5	1.1	(0.52, 2.33)	0.8	0.13	
Vomiting	Double-arm meta-analysis, random effect model	-	Total	9	1.1	(0.61, 1.98)	0.76	0.06	Zhang et al. 2021 [37]
			Sensitivity analysis	8	1.59	(1.16, 2.19)	0.004	0.44	
Hallucinations	Double-arm meta-analysis, fixed effect model	-	Total	6	6.68	(1.99, 22.37)	0.002	0.98	
Headache	Double-arm meta-analysis, fixed effect model	-	Total	4	1.95	(0.77, 4.98)	0.16	0.73	
Diplopia	Double-arm meta-analysis, fixed effect model	-	Total	3	1.66	(0.56, 4.95)	0.36	0.37	
Blurred vision	Double-arm meta-analysis, fixed effect model	-	Total	3	5.2	(1.33, 20.37)	0.02	0.38	
Dizziness	Double-arm meta-analysis, random effect model	-	Total	14	1.9	(1.28, 2.82)	0.001	0.008	Shen et al. 2023 [29]
			Sensitivity analysis	13	1.69	(1.25, 2.30)	0.0007	0.14	

**Fig. 4:**
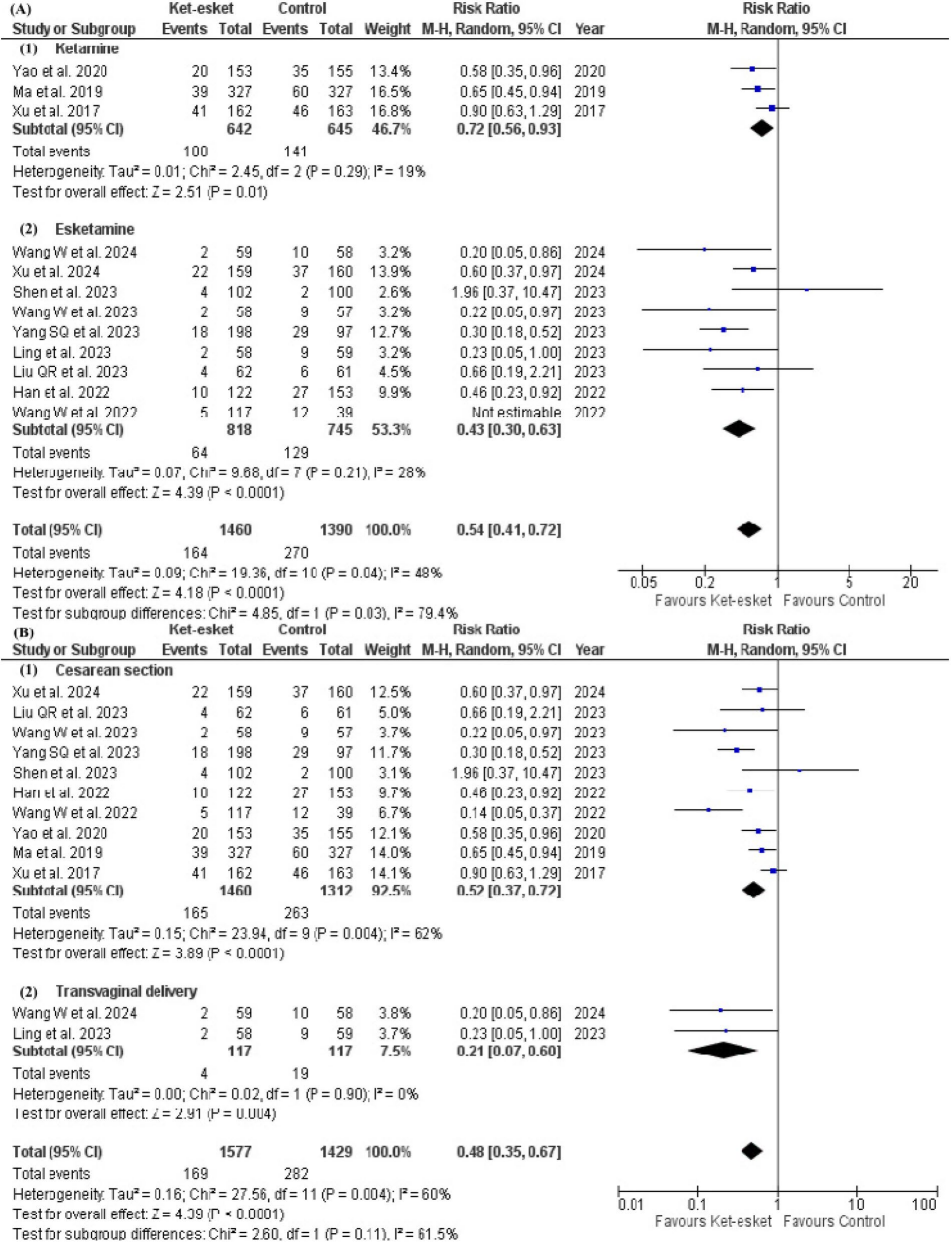
Forest plot of (**A**) subgroup analysis according to the nature of drug for the short-term occurrence of PPD, (**B**) subgroup analysis according to the mode of delivery for the short-term occurrence of PPD

Our subgroup analysis based on the nature of intervention concluded a significant difference between ketamine and esketamine subgroups with a *p*-value of 0.01 in short-term PPD (ketamine: RR = 0.72, 95% CI [0.56, 0.93], *P* = 0.01; esketamine: RR = 0.38, 95% CI [0.25, 0.57], *P* < 0.00001). Heterogeneity was resolved in the ketamine subgroup, but pooled studies in the esketamine subgroup remained heterogeneous (ketamine: I² =19%, *P* = 0.29; esketamine: I² =45%, *P* = 0.07). However, heterogeneity was resolved in the esketamine subgroup after leaving out Wang W et al. 2022 (I² =28%, *P* = 0.21), and the effect estimate of the subgroup favoured the esketamine group (RR = 0.43, 95% CI [0.30. 0.63], *P* < 0.0001) (Fig. 4A) (Table 3).

Additionally, the Subgroup analysis based on the mode of delivery in the short-term PPD is presented in Fig. 4B; Table 3. The estimates of the caesarean section subgroup favoured the Ket-esket group over the control group (RR = 0.52, 95% CI [0.37, 0.72]) in the occurrence of short-term PPD. However, heterogeneity remained unresolved in the caesarean section subgroup even after conducting a leave-one-out test.

**Fig. 5:**
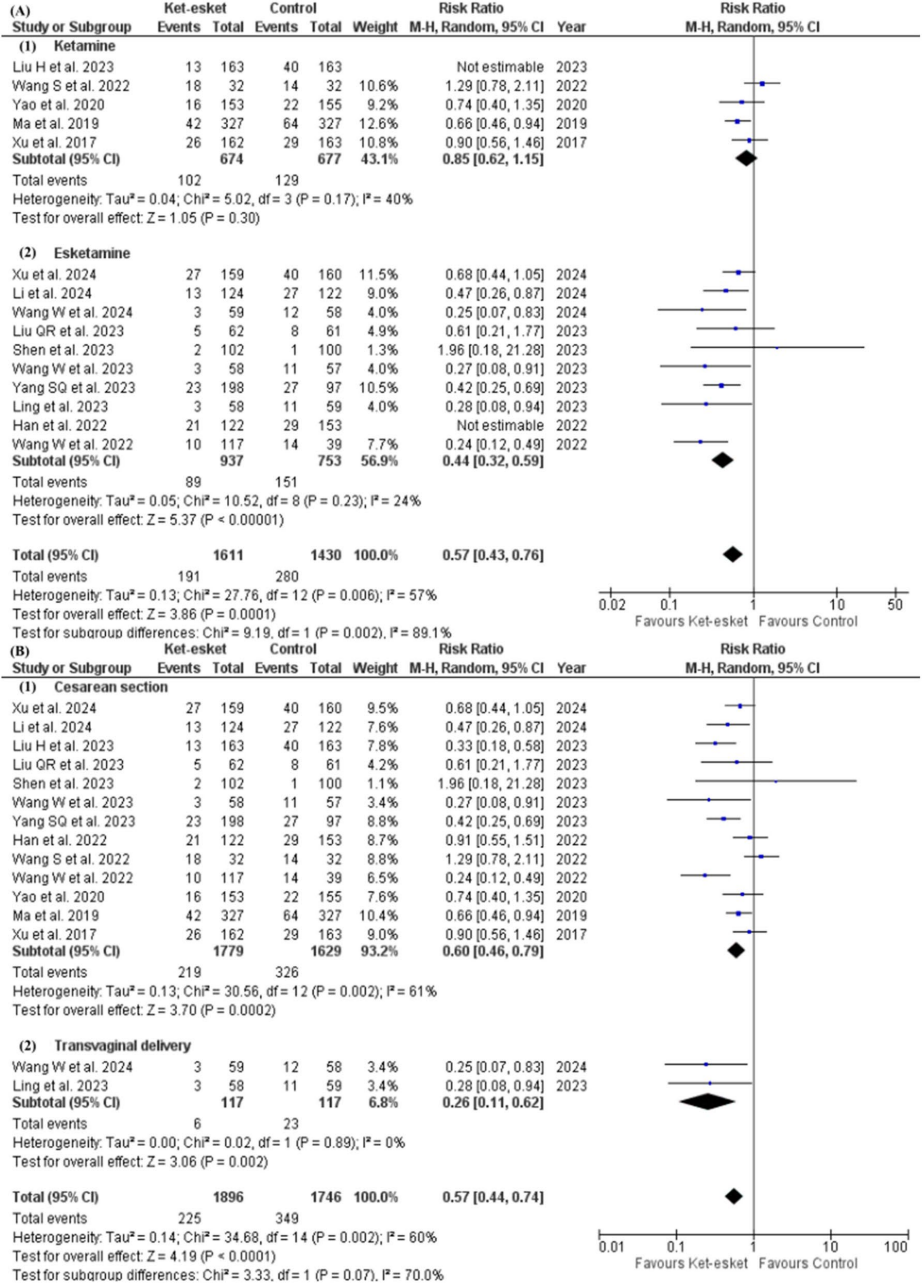
Forest plot of (**A**) subgroup analysis according to the nature of drug for the long-term occurrence of PPD, (**B**) subgroup analysis according to the mode of delivery for the long-term occurrence of PPD

Also, subgrouping based on the nature of intervention in long-term PPD concluded no significant difference between ketamine and esketamine subgroups, but the pooled risk ratio in the ketamine subgroup did not favour either ketamine or control (ketamine: RR = 0.72, 95% CI [0.48, 1.09], *P* = 0.12; esketamine: RR = 0.48, 95% CI [0.35, 0.67], *P* < 0.0001). Heterogeneity remained unresolved in both subgroups (ketamine: I² =71%, *P* = 0.008; esketamine I² =45%, *P* = 0.06) (Fig. 3D) (Table 3). Leave-one-out test resolved heterogeneity in each subgroup separately after the exclusion of Liu H et al. 2023 and Han et al. 2022 from ketamine and esketamine subgroups, respectively (Ketamine: I² =40%, *P* = 0.17; esketamine: I² =24%, *P* = 0.23) Fig. (5 A). Effect estimates are reported in the analysis summary in Table 3.

Additionally, the subgroup analysis is based on the long-term mode of delivery, as presented in Fig. 5B; Table 3. The estimates of the caesarean section subgroup favoured the Ket-esket group over the control (RR = 0.60, 95% CI [0.46, 0.79], *P* = 0.002) long-term PPD. However, heterogeneity remained unresolved in the caesarean section subgroup even after conducting a leave-one-out test.

**Fig. 6:**
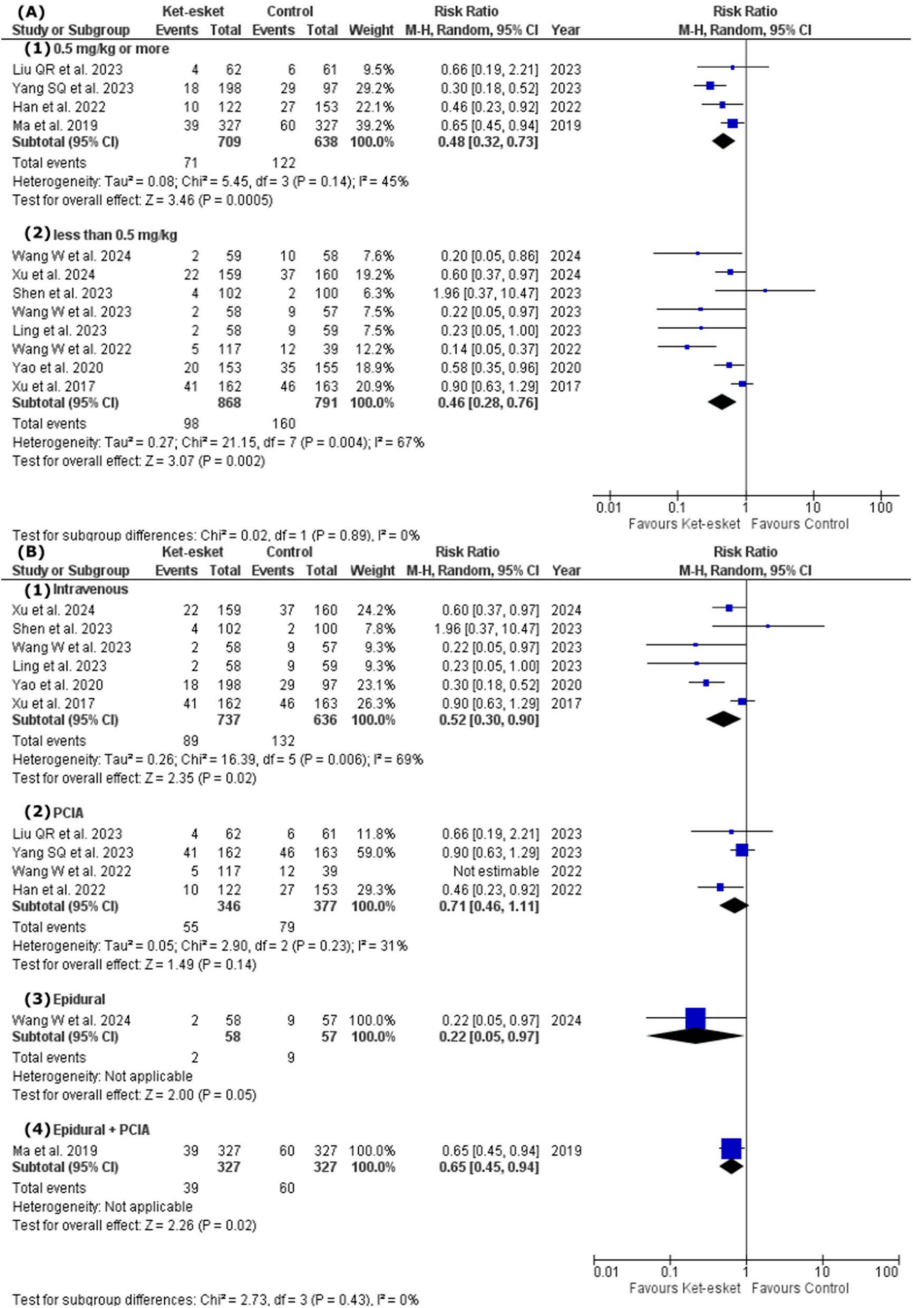
Forest plot of (**A**) subgroup analysis according to the dose of the drug for the short-term occurrence of PPD, (**B**) subgroup analysis according to the route of administration for the short-term occurrence of PPD

A subgroup analysis based on intervention dose concluded the efficacy of high dose subgroup (0.5 mg/kg or more) and low dose subgroup (less than 0.5 mg/kg) in short-term PPD. (high dose: RR = 0.48, 95% CI [0.32, 0.73], *P* = 0.0005; low dose: RR = 0.46, 95% CI [0.28, 0.76], *P* = 0.002). Heterogeneity was resolved in the high-dose subgroup but remained unresolved in the low-dose subgroup (high dose: I² =45%, *P* = 0.14; low dose: I² =67%, *P* = 0.004). Heterogeneity was not resolved in the low-dose subgroup after removing a single study in each scenario (Fig. 6A) (Table 3). The subgroup analysis is based on the short-term administration route and is presented in Fig. 6B; Table 3. The heterogeneity was resolved in the PCIA subgroup after excluding Wang W et al., 2022 from the subgroup (I² =31%, *P* = 0.23). However, the effect estimate did not favour either of the two groups in the PCIA subgroup (RR = 0.71, 95% [0.46, 1.11], *P* = 0.14). Heterogeneity remained high in the rest of the subgroups.

**Fig. 7:**
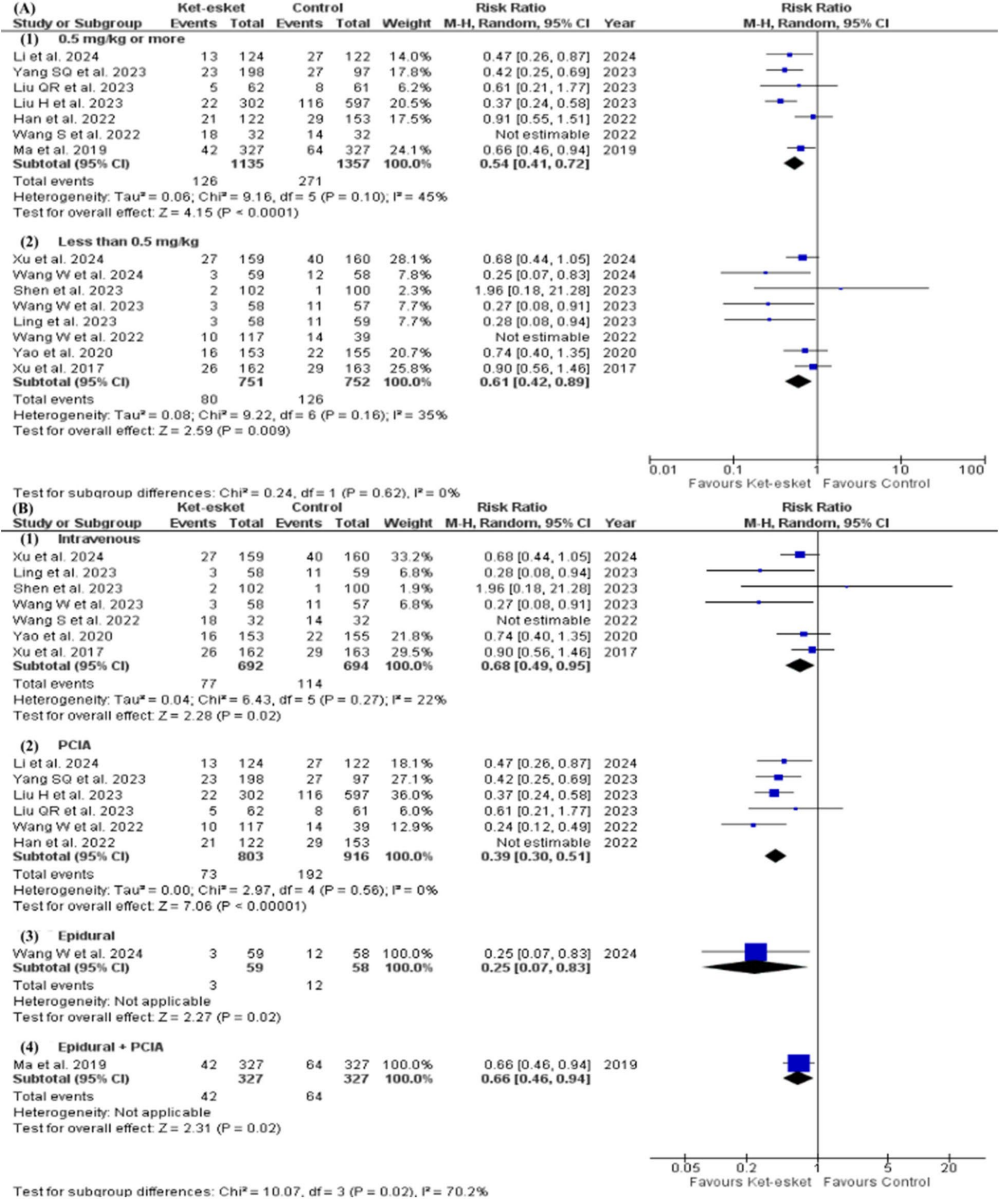
Forest plot of (**A**) subgroup analysis according to the dose of the drug for the long-term occurrence of PPD, (**B**) subgroup analysis according to the route of administration for the long-term occurrence of PPD

Also, subgrouping based on dose in long-term PPD concluded no significant difference between high-dose and low-dose subgroups (high dose: RR = 0.62, 95% CI [0.44, 0.88], *P* = 0.008; low dose: RR = 0.51, 95% CI [0.33, 0.78], *P* = 0.002). Heterogeneity remained unresolved in both subgroups (high dose: I² =69%, *P* = 0.004; low dose: I² =56%, *P* = 0.03). However, heterogeneity was resolved in both high-dose and low-dose subgroups after excluding Wang S et al., 2022 and Wang W et al., 2022 from each subgroup respectively (high dose: I² =45%, *P* = 0.10; low dose: I² =35%, *P* = 0.16) (Fig. 7A) (Table 3). The subgroup analysis is based on the long-term administration route and is presented in Fig. (7B) and Table 3. The heterogeneity was resolved in both intravenous and PCIA subgroups after the exclusion of Wang S et al., 2022 and Han et al., 2022, respectively (intravenous: I² =22%, *P* = 0.27; PCIA: I² =0%, *P* = 0.56). The effect estimates favoured the ket-esket group over the control in both subgroups (intravenous: RR = 0.68, CI [0.49, 0.95], *P* = 0.02); PCIA: RR = 0.39, CI [0.30, 0.51], *P* < 0.00001).

To explore sources of heterogeneity, meta-regression was performed regarding both patients’ ages and ketamine/esketamine doses. Meta-regression indicated no significant correlation between short-term PPD and age (*P* = 0.102) or dose of intervention (*P* = 0.447). Also, a regression model indicated no significant correlation between long-term PPD and intervention dose (*P* = 0.673). In contrast, a significant positive correlation between long-term PPD and age was identified in a regression model (*P* = 0.002, *ρ* = 0.242).

#### EPDS

Regarding short-term EPDS, A total of nine studies [16, 22, 23, 27, 28, 35, 37, 39, 40] were analysed in this outcome. The overall mean difference favoured the ketamine/esketamine group over the control group (MD=−0.98, 95% CI [−1.36, −0.59], *P* < 0.00001). Heterogeneity was high (*P* = 0.002, I² =67%) between studies. Sensitivity analysis did not resolve heterogeneity between the pooled studies (supplementary Fig. 2A) (Table 3).

Concerning long-term EPDS, Pooling results from ten studies [15, 16, 22–24, 27, 28, 35, 39, 40] favoured the ketamine/esketamine group over the control group (MD=−1.03, 95% CI [−1.62, −0.46], *P* = 0.0005). High heterogeneity was observed between pooled studies (*P* < 0.00001, I²=87%). Heterogeneity remained unresolved after removing a single study in multiple scenarios (supplementary Fig. 2B) (Table 3).

#### Short-term EPDS

In a subgroup analysis for short-term EPSD based on nature of intervention, heterogeneity remained high in both ketamine and esketamine subgroups (ketamine: *P* = 0.002, I² =80%); esketamine: *P* = 0.002, I² =67 with no significant difference between the two subgroups (ketamine: MD=−1.04, 95% CI [−1.93, −0.16], *P* = 0.02; esketamine: MD=−0.99, 95% CI [−1.36, −0.58], *P* < 0.00001). However, heterogeneity was resolved in both subgroups after excluding Monks et al. 2022 and Han et al. 2022 from the ketamine and esketamine subgroups, respectively (Ketamine: I² =26%, *P* = 0.26; esketamine: I² =1%, *P* = 0.39). The overall mean difference favoured ketamine or esketamine in both subgroups. Effect estimates are reported in supplementary Fig. 3A and Table 3.

Another subgrouping for short-term EPSD based on intervention dose was conducted and concluded efficacy in reducing EPDS scores for both high dose and low dose subgroups (high dose: MD= −1.44, 95% CI [−2.26, −0.62], *P* = 0.0005; low dose: MD=−0.71, 95% CI [−1.05, −0.37], *P* < 0.0001). Heterogeneity was resolved in the low-dose subgroup (*P* = 0.22, I² =30%). However, studies in the high-dose subgroup remained heterogeneous (*P* = 0.001, I² =81%) even after conducting the leave-one-out tests in the high-dose group separately. Effect estimates are reported in supplementary Fig. 3B and Table 3.

Additionally, a subgroup analysis was conducted for short-term EPSD based on the route of administration. Heterogeneity remained high among subgroups of different routes of administration. However, heterogeneity was resolved in the intravenous route subgroup after excluding Monks et al., 2022 from the subgroup (I²=48%, *P* = 0.13). Different routes of administration significantly favoured the ketamine/esketamine group over the control group. Effect estimates are reported in supplementary Fig. 3C and Table 3.

Six studies were included in this meta-analysis model regarding the change from baseline in short-term EPDS. The overall mean difference favoured the intervention group over the control group (MD=−1.21, 95% CI [−2.31, −0.29], *P* = 0.01. Heterogeneity was high (*P* = 0.0002, I² =80%) between studies. Heterogeneity was not resolved after removing a single study from the meta-analysis model in multiple scenarios. Results of a subgroup analysis based on the nature of the intervention are presented in supplementary Fig. (4 A-B) and Table 3.

#### Long-term EPDS

An insignificant difference between ketamine and esketamine subgroups was concluded in a subgroup analysis based on the nature of the intervention (ketamine: MD=−1.45, 95% CI [−2.62, −028], *P* = 0.02; esketamine: MD=−0.87, 95% CI [−1.58, −0.17], *P* = 0.02). Heterogeneity remained unresolved in both subgroups (ketamine: *P* < 0.00001, I²=88%; esketamine: *P* < 0.00001, I² =87%). Effect estimates are reported in supplementary Fig. 5A and Table 3.

Our subgroup analysis based on intervention dose concluded efficacy for both high-dose and low-dose subgroups in lowering long-term EPDS scores (high dose: MD=−1.60, 95% CI [−2.66, −0.54], *P* = 0.003; low dose: MD=−0.55, 95% CI [−1.02, −0.08], *P* = 0.02). Pooled studies remained heterogeneous in both subgroups (high dose: *P* < 0.00001, I² =92%; low dose: *P* = 0.06, I² =60%). However, heterogeneity was resolved in the low-dose group after excluding Wang Y et al. 2022 from the subgroup (I² =0%, *P* = 0.46). Leave-one-out test did not resolve heterogeneity in the high-dose subgroup. Effect estimates are reported in supplementary Fig. 5B and Table 3.

Additional subgroup analysis was conducted based on the route of administration. Heterogeneity was not resolved among subgroups of different routes of administration. Different routes of administration significantly favoured the ketamine/esketamine group; estimates and *P* values are reported in supplementary Fig. (5 C) and Table 3.

Seven studies were pooled in this meta-analysis model regarding the change from baseline in long-term EPDS. The overall estimate of pooled studies favoured the ketamine/esketamine group over the control group (MD=−0.86, 95% CI [−1.47, −0.26], *P* = 0.005). Pooled studies were not homogenous (*P* = 0.02, I² =62%). Heterogeneity was best resolved after omitting Guo et al., 2023 from the meta-analysis model (*P* = 0.31, *I²* =16%). The overall estimate after removing Guo et al., 2023 still favoured the intervention group (MD=−1.07, 95% CI [−1.53, −0.61], *P* < 0.00001). A subgroup analysis based on the nature of the intervention was conducted, and results are demonstrated in supplementary Fig. (6 A-B) and Table 3.

### Pain score

The standardised mean difference was adopted as an estimate in two pooling analyses of day 1 and day 2-3 pain scores. The overall estimate of the two pooling analyses favoured ketamine/esketamine over the control group (day 1 pain: SMD=−1.15, 95% CI [−1.69, −0.62], *P* < 0.0001; day 2–3 pain: SMD=−1.31, 95% CI [−1.87, −0.75], *P* < 0.00001]. Both meta-analysis models had high heterogeneity (*P* < 0.00001, I² =96%).

Heterogeneity remained unresolved in esketamine subgroups after a subgroup analysis based on the nature of intervention of both analyses. However, it was resolved in the ketamine subgroup of day 2-3 pain score with two studies only in the subgroup.

In the day 1 pain score, heterogeneity was resolved in the high-dose group (*P* = 0.16, I² =42%). In contrast, heterogeneity remained high in the low-dose subgroup (*P* < 0.00002, I² =98%). Studies in the pain score for days 2-3 remained heterogeneous in both high-dose and low-dose subgroups.

A subgroup analysis was conducted based on routes of administration in both day 1 and day 2-3 pain scores. Heterogeneity remained unresolved in different routes of administration in both analyses. However, heterogeneity was resolved in PCIA subgroups of both day 1 and day 2-3 pain after removing Li et al. 2024 and Guo et al. 2023, respectively. Intravenous and PCIA routes significantly favoured the ketamine/esketamine group over the control group. In contrast, the subcutaneous route subgroup incorporating only Monks et al. 2022 favoured the control group over the ketamine/esketamine group in the day 1 pain score but did not favour either of the two groups in the day 2-3 pain score.

Additional subgroup analysis was conducted based on the mode of delivery in day 1 pain score. All included studies in the meta-analysis model of day 2-3 pain scores were meant to investigate patients who underwent caesarean section. The estimate of the caesarean section subgroup significantly favoured the ketamine/esketamine group over the control group (MD=−1.16, 95% CI [−1.74, −0.58], *P* < 0.0001), and heterogeneity remained unresolved in the subgroup (*P* < 0.00001, I² =96%). Results are demonstrated in Table 3 and supplementary Figs. (7–9).

### Side effects

#### Dizziness

Dizziness as a side effect was reported in 14 studies [16, 22–24, 26–31, 34, 35, 37, 40]. The overall risk ratio (RR) favoured the control group over the ketamine group (RR 1.90, 95% CI [1.28, 2.28], *P* = 0.001). Pooled studies were not homogenous (*P* = 0.008, I² =54%). A sensitivity analysis was conducted in order to solve heterogeneity by excluding one study in each scenario. Heterogeneity was best resolved by excluding the study of Shen et al., 2023 (*P* = 0.14, I-square = 30%). After removing Shen et al. [29] from the meta-analysis model, the overall risk ratio still favoured the control group over the ketamine group (RR 1.69, 95% CI [1.25, 2.30], *P* = 0.0008), as illustrated in Table 3 and supplementary Fig. 10A.

A meta-regression model was conducted in order to explore the relationship between the age of patients and the dose of ketamine/esketamine with dizziness. The regression model concluded no significant correlation (*P* = 0.135, *P* = 0.185) for both age and dose respectively.

#### Nausea

This meta-analysis model included 6 studies [23, 26, 28, 29, 36, 37]. The overall risk ratio did not favour either ketamine/esketamine or control groups (RR = 0.83, 95% CI [0.32, 2.16], *P* = 0.70). Pooled studies were not homogenous (*P* = 0.02, I² =64%). Heterogeneity was best resolved by excluding the study of Zhang et al. 2021 [37], (*P* = 0.13, I² =44%). After omitting Zhang et al. from the meta-analysis model, the overall risk ratio still did not favour either of the two groups (RR = 1.10, 95% CI [0.52, 2.33], *P* = 0.80), as demonstrated in Table 3 and supplementary Fig. 10B.

#### Vomiting

Nine studies were pooled in this analysis [16, 23, 26–29, 35–37]. The overall risk ratio did not favour either ketamine/esketamine or control groups (RR = 1.10 95% CI [0.61 1.98]*P* = 0.76). Pooled studies were not homogenous (*P* = 0.06, I² =47%). Heterogeneity was best resolved by excluding the study of Zhang et al., 2021 (*P* = 0.44, I² =0%). After removing Zhang et al. [37] from the meta-analysis model, the overall risk ratio favoured the control group (RR = 1.59, 95% CI [1.16 to 2.19], *P* = 0.004), as illustrated in Table 3 and supplementary Fig. 10C.

#### Headache

Four studies [16, 23, 29, 35] were included in this meta-analysis model. The overall risk ratio between the ketamine/esketamine group did not favour either of the two groups (RR = 1.95, 95% CI [0.77, 4.98], *P* = 0.16). Pooled studies were homogenous (*P* = 0.73, I²=0%), as demonstrated in Table 3 and supplementary Fig. 11A.

#### Blurred vision

The overall risk ratio of the three pooled studies [28, 29, 34] favoured the control group over the ketamine/esketamine group (RR = 5.20, 95% CI [1.33, 20.37], *P* = 0.02). No heterogeneity was observed between the pooled studies (*P* = 0.38, I² =0%). Zhang et al. [37] reported no events of blurred vision in both arms, as illustrated in Table 3 and supplementary Fig. 11B.

#### Hallucinations

We included six studies in this model [16, 26, 27, 30, 34, 35]. The overall risk ratio between the ketamine/esketamine and control groups favoured the control group (RR = 6.68, 95% CI [1.99, 22.37], *P* = 0.002). Pooled studies were homogenous (*P* = 0.98, I² = 0%). Guo et al. [38], Shen et al. [29], and Monks et al. [28] reported no events of hallucination in both ketamine and control groups, as shown in Table 3 and supplementary Fig. 11C.

#### Diplopia

Three studies [28, 30, 35] were included in this meta-analysis model. The overall risk ratio did not favour either the ketamine/esketamine group or the control group (RR = 1.66, 95% CI [0.56, 4.95], *P* = 0.36). The meta-analysis model showed no heterogeneity between the pooled studies (*P* = 0.37, I² =0%). Guo et al. 2023 [22], Shen et al. 2023 [29] and Wang W 2022 [30] reported no events of diplopia in both arms, as illustrated in Table 3 and supplementary Fig. 11D.

Estimates, confidence intervals, and *P* values are presented in the summary of our meta-analysis (Table 3). Data of different outcomes that were considered uninformative for meta-analysis are summarised in supplementary Table 2.

## Discussion

In this systematic review and meta-analysis, we addressed the potential effect of ketamine and Esketamine on PPD after caesarean or vaginal delivery. Several studies have reported that ketamine and esketamine are efficient in the treatment of several depressive disorders which suggests their potential efficacy in the prevention of PPD [41–44].

Ketamine was approved by the FDA in the year nineteen seventy as an anaesthetic drug. At the dawn of the 21st century, ketamine unfolded a new revelation of its potential when Berman et al. first reported its antidepressant effect in patients with MDD. The mechanism of action of ketamine as an antidepressant has not been fully expounded. However, it’s postulated to its antagonistic properties on NMDA receptors of GABA interneurons and its agonistic properties on AMPA receptors [45, 46].

We found that ketamine and esketamine significantly decreased the incidence of short-term and long-term PPD when compared to the control group. The meta-analysis conducted by Li et al., 2024 which included women who underwent caesarean delivery, came in agreement with our results. They found that Ketamine and esketamine significantly lowered the risk ratio of long-term and short-term PPD among women when compared to the control group (*P* values were 0.0006 and *<* 0.0001, respectively) [47].

In our study, when subgroup analysis was performed for ketamine and esketamine separately compared to the control group, we found a significant effect for each drug in reducing the incidence of short-term PPD. In contrast, only esketamine was significantly effective in reducing the incidence of short-term PPD. In contrast to our results, Li et al., 2024 found that only esketamine showed a significant decrease in the incidence of PPD on the short-term and long-term levels (*p*-value = 0.007 and 0.02 respectively) [44]. This may be attributed to the potent and rapid antidepressant effect of esketamine compared to ketamine. It was found that esketamine is 3 times more potent than esketamine since it has more affinity to the NMDA receptors [48].

In this study, Subgroup analysis based on the route of administration revealed a significant efficacy for Ketamine/esketamine in reducing the incidence of short-term PPD when administered through intravenous, epidural, or (epidural + PCIA) routes. However, no significant effect was observed for the PCIA route alone. All the routes were significantly effective in lowering the incidence of long-term PPD except the intravenous route. Likewise, the study conducted by Li et al., 2022 among women following caesarean delivery reported that intravenous ketamine significantly lowered the incidence of short-term PPD as well as the PPD scores in comparison to the control group (*P* values = 0.0007 and 0.002 respectively). They also reported that there was no significant difference between the groups in the long-term PPD scores [49]. Moreover, the Ma et al. meta-analysis that included women after caesarean delivery found that intravenous esketamine is significantly effective in reducing the incidence of short-term PPD (*P* value < 0.0001), whereas no significant effect was observed on the long-term PPD (*P* value = 0.14) [50].

In addition, we performed subgroup analysis according to the dose, which revealed the efficacy of doses less than 0.5 mg and doses of 0.5 mg ketamine/esketamine in lowering the incidence of long-term and short-term PPD. On the contrary, Li et al., 2024 found that only high doses efficiently reduced the incidence of short-term and long-term PPD (*p*-value < 0.0001 and 0.002, respectively [47].

Regarding the risk of side effects, patients in the Ketamine/esketamine group showed statistically significant higher rates of developing blurred vision, dizziness, hallucinations and headache than women in the control group. However, no significant difference was observed between the groups in Nausea, vomiting, and Diplopia. Li et al., 2024 have similar findings regarding the risk of side effects, but the risk of diplopia was significantly higher among the Ketamin/esketamine group (*p*-value = 0.01) [47].

Despite the fact that the reported side effects are temporary and usually resolve after discontinuation of the drugs, we recommend using small doses [51]. Since low doses were as efficient as high doses, this study suggests using the lowest possible dose to be more tolerable and avoid the reported side effects.

Regarding short-term and long-term EPDS scores, both Ketamine and esketamine showed significantly lower scores than women in the control group. Li et al., 2024 found that only esketamine significantly lowered short-term and long-term EPDS scores. Also, they found significant results only with the PCIA route of administration and high doses of the drugs. Whereas, in our study, we found significant results for high and low doses as well as PCIA and IV routes of administrations [47]. We also found that only esketamine effectively improved the change in short-term EPDS score from baseline. However, both of the drugs significantly affected the change in long-term EPDS score from baseline.

Moreover, ketamine and esketamine are effective in both modes of delivery, either caesarean or vaginal, when compared to a control group, based on a subgroup analysis of the studies included in this meta-analysis. Research has found that there is no significant difference between caesarean and normal delivery in the risk of PPD [52–54]. History of emesis during pregnancy, previous depression, and being a housewife have been identified as risk factors for PPD. Thus, women with these risk factors need special care [55].

Our study is a comprehensive systematic review and meta-analysis dealing with a large population with a diversity of doses and routes of administration. The comprehensiveness of our systematic review allowed us to conduct various subgroup analyses to deal with such diversity. The subgroup analyses allowed us to examine the effect of ket/esket on postpartum depression delicately. Most of the included studies were randomised controlled trials of high quality depending on the RoB2 quality assessment tool. The number of studies allowed us to perform meta-regression and investigate sources of heterogeneity in some outcomes.

Including both experimental and observational designs in a meta-analysis model allowed for a more comprehensive result. However, it poses a significant challenge as observational designs are more prone to bias. Another challenge is the inconsistent reporting of outcomes between the two study designs. We performed a thorough quality assessment of the included studies using RoB2 and NOS tools. We followed a careful outcome selection process to overcome the potential biases attributed to the variability of study designs.

The subgroup analyses conducted in this study didn’t solve most of the heterogeneity, which made a significant limitation of this study. However, whenever feasible, the leave-one-out test was conducted after subgroup analysis in each subgroup separately. Leave-one-out test of subgroups resolved heterogeneity in many subgroups. Heterogeneity might be attributed to the differences in doses, routes of administration, and mode of delivery (emergency caesarean, elective caesarean, and normal delivery). This study was incapable of controlling the impact of unmeasured confounding variables, taking into consideration its design. This might be the source of unresolved heterogeneity in many models, limiting our results’ generalizability. However, meta-regression models were conducted whenever feasible to investigate possible confoundings and sources of heterogeneity.

Also, the noted publication bias poses a challenge for such meta-analysis due to the potential risk of overestimating the true effect size as a result of the possible loss of negative results. In addition, there was no diversity in the studied sample, where 19 out of the 21 included studies had Chinese populations. Thus, further trials are needed in different regions of the world to address the efficacy and safety of esketamine among a diverse population refraining from the possible underreporting of studies with certain types of results. Further well-structured clinical trials will facilitate the development of stronger meta-regression models.

In conclusion, Ketamine and esketamine are effective in lowering the incidence of occurrence of short-term PPD. On the other hand, only esketamine is effective in reducing the incidence of long-term PPD. Epidural or epidural + PCIA are effective routes for both long-term and short-term development of PPD. The drugs don’t have long-term serious side effects. However, temporary side effects such as dizziness, vomiting, blurred vision and hallucinations were reported. Moreover, doses less than 0.5 mg and those of 0.5 mg or more were both significantly effective in comparison to the control groups. Thus, it is recommended to use smaller doses for a more tolerable treatment period without anxious side effects.

## Supplementary Information

Supplementary Material 1.

Supplementary Material 2.

## Data Availability

No datasets were generated or analysed during the current study.

## Abbreviations

PPD: Postpartum depression
DSM-5: Disorders-Fifth Edition
MDD: Major depressive disorder
AD: Antidepressants
NMDA: N-methyl-D-aspartate
ICD: International Classification of Disease
BMI: Body mass index
EPDS: Edinburgh Postnatal Depression Scale
