# Supplementary material for: Efficacy and safety of ketamine and esketamine in reducing the incidence of postpartum depression: an updated systematic review and meta-analysis
Source: BMC Pregnancy Childbirth. 2025 Feb 6;25:125. doi: 10.1186/s12884-025-07186-y (PMC11800651; doi:10.1186/s12884-025-07186-y)
Supplement: Supplementary file 2 — Supplementary Material 2. [file 12884_2025_7186_MOESM2_ESM.docx]

Supplementary tables:

Full Search strategy.

| **PubMed/Medline** | |
| --- | --- |
| #1 | (Ketamine OR S-Ketamine OR esketamine OR "2-(2-Chlorophenyl)-2-(methylamino)cyclohexanone" OR CI-581 OR "CI 581" OR CI581 OR Ketalar OR Ketaset OR Ketanest OR Calipsol OR Kalipsol OR Calypsol OR narkamon OR keta OR ketmin OR ketava OR ketalin OR ketina OR brevinaze OR keta‐hameln OR Ketamines OR Spravato OR Ketalar OR Eskesia OR Ketanest-S OR Keta-S OR "Ketamine"[Mesh]) |
| #2 | (postpartum OR Post-Partum OR "Post Partum" OR Puerperium OR puerperal OR postnatal OR Post-Natal OR "Post Natal" OR "fourth trimester" OR childbirth OR delivery OR "Postpartum Period"[Mesh]) |
| #3 | (Depressive OR Depression OR Depressions OR Dysphoria OR "mood disorder" OR "Adjustment Disorder" OR "Affective Disorder" OR "Affective Symptoms" OR depressed OR "Depression"[Mesh]) |
| #4 | (EPDS OR "edinburgh postnatal depression scale") |
| #5 | #1 AND ((#2 AND #3) OR #4) |
| **Cochrane CENTRAL** | |
| #1 | (Ketamine OR S-Ketamine OR esketamine OR "2-(2-Chlorophenyl)-2-(methylamino)cyclohexanone" OR CI-581 OR "CI 581" OR CI581 OR Ketalar OR Ketaset OR Ketanest OR Calipsol OR Kalipsol OR Calypsol OR narkamon OR keta OR ketmin OR ketava OR ketalin OR ketina OR brevinaze OR keta‐hameln OR Ketamines OR Spravato OR Ketalar OR Eskesia OR Ketanest-S OR Keta-S). |
| #2 | (postpartum OR Post-Partum OR "Post Partum" OR Puerperium OR puerperal OR postnatal OR Post-Natal OR "Post Natal" OR "fourth trimester" OR childbirth OR delivery) |
| #3 | (Depressive OR Depression OR Depressions OR Dysphoria OR "mood disorder" OR "Adjustment Disorder" OR "Affective Disorder" OR "Affective Symptoms" OR depressed) |
| #4 | (EPDS OR "edinburgh postnatal depression scale") |
| #5 | #1 AND ((#2 AND #3) OR #4) |
| **Web of Science** | |
| #1 | (Ketamine OR S-Ketamine OR esketamine OR "2-(2-Chlorophenyl)-2-(methylamino)cyclohexanone" OR CI-581 OR "CI 581" OR CI581 OR Ketalar OR Ketaset OR Ketanest OR Calipsol OR Kalipsol OR Calypsol OR narkamon OR keta OR ketmin OR ketava OR ketalin OR ketina OR brevinaze OR keta‐hameln OR Ketamines OR Spravato OR Ketalar OR Eskesia OR Ketanest-S OR Keta-S) (Topic) |
| #2 | (postpartum OR Post-Partum OR "Post Partum" OR Puerperium OR puerperal OR postnatal OR Post-Natal OR "Post Natal" OR "fourth trimester" OR childbirth OR delivery) (Topic)   (Topic) |
| #3 | (Depressive OR Depression OR Depressions OR Dysphoria OR "mood disorder" OR "Adjustment Disorder" OR "Affective Disorder" OR "Affective Symptoms" OR depressed) (Topic) |
| #4 | (EPDS OR "edinburgh postnatal depression scale") (Topic) |
| #5 | #1 AND ((#2 AND #3) OR #4) |
| **Scopus** | |
| TITLE-ABS-KEY (Ketamine OR S-Ketamine OR esketamine OR "2-(2-Chlorophenyl)-2-(methylamino)cyclohexanone" OR CI-581 OR "CI 581" OR CI581 OR Ketalar OR Ketaset OR Ketanest OR Calipsol OR Kalipsol OR Calypsol OR narkamon OR keta OR ketmin OR ketava OR ketalin OR ketina OR brevinaze OR keta‐hameln OR Ketamines OR Spravato OR Ketalar OR Eskesia OR Ketanest-S OR Keta-S)  AND  (((postpartum OR Post-Partum OR "Post Partum" OR Puerperium OR puerperal OR postnatal OR Post-Natal OR "Post Natal" OR "fourth trimester" OR childbirth OR delivery) AND (Depressive OR Depression OR Depressions OR Dysphoria OR "mood disorder" OR "Adjustment Disorder" OR "Affective Disorder" OR "Affective Symptoms" OR depressed)) OR (EPDS OR "edinburgh postnatal depression scale")) | |

Table 1. Detailed search strategy for each database

| Study | Year | Selection | | | | Comparability | | Outcome | | | Quality |
| --- | --- | --- | --- | --- | --- | --- | --- | --- | --- | --- | --- |
|  |  | Representativeness of the exposed | Non-  exposed | Ascertainment of exposure | Begin without outcome present |  |  | Outcome assessment | Follow-up length | Adequacy  of outcome |  |
| Liu H | 2023 | * | * | * | * | * | * | | * | * | Good |
| Lou | 2023 | * | * | * | * | ** | * | | * | * | Good |
| Wang Y | 2022 | * | * | * | * | * | * | | * | * | Good |

Table 2. Newcastle-Ottawa Scale (NOS) quality assessment summary for included studies

| **Study ID** | **Group** | **Short-term depression score** | **Long-term depression score** | **Day 1 postopartum pain** | **Day 2 postopartum pain** | **Event of nausea and vomiting** | **N** |
| --- | --- | --- | --- | --- | --- | --- | --- |
| **Shen 2023** | IV Ketamine 0.25 mg/kg | 2.25 (0-17) | 1.36 (0-18) | 1 (0-3) | 0 (0-2) |  | 100 |
|  | Control | 2.4 (0-14) | 1.4 (0-13) | 1 (0-4) | 0 (0-4) |  | 102 |
| **Wang S 2022** | IV ketamine 0.5 mg/kg | 9 [6-13] | 10 [7-12] | 4 [3-7] |  | 7 (21.2) | 33 |
|  | Control | 8 [6-10] | 9 [7-11] | 4 [3-4] |  | 5 (15.2) | 33 |
| **Wang W 2022** | IV ketamine 0.4 mg/kg |  |  |  |  | 3 (7.7) | 39 |
|  | IV Ketamine 0.2 mg/kg |  |  |  |  | 4 (10.0) | 40 |
|  | IV Ketamine 0.1 mg/kg |  |  |  |  | 4 (10.5) | 38 |
|  | Control |  |  |  |  | 11 (28.2) | 39 |
| **Xu 2017** | IV ketamine 0.25 mg/ kg |  |  |  | 4 (0-7) |  | 162 |
|  | Control |  |  |  | 4 (0-8) |  | 163 |
| **Wang W 2023** | IV Ketamine 0.2 mg/kg |  |  |  |  | 3 (5.2) | 58 |
|  | Control |  |  |  |  | 4 (7.0) | 57 |
| **Xu 2024** | Esketamine 0.2 mg/kg |  |  |  |  | 12 (7.5) | 159 |
|  | Control |  |  |  |  | 13 (8.1) | 160 |
| **Wang W 2024** | Esketamine 0.2 mg/kg |  |  |  |  | 2 (3.4) | 59 |
|  | Control |  |  |  |  | 3 (5.2) | 58 |
| **Li 2024** | Esketamine 1.5 mg/kg |  |  |  |  | 34 (2.4) | 124 |
|  | Control |  |  |  |  | 31 (25.4) | 122 |
| **Ling 2023** | Esketamine 0.2 mg/kg |  |  |  |  | 2 (3.4) | 58 |
|  | Control |  |  |  |  | 1 (1.7) | 59 |
| **Guo 2023** | Esketamine 1 mg/kg |  |  |  |  | 5 (5.9) | 85 |
|  | Control |  |  |  |  | 13 (15.2) | 85 |
| **Wang Y 2022** | Esketamine 0.35 mg/kg |  |  |  |  | 27 (25) | 108 |
|  | Control |  |  |  |  | 26 (19.7) | 132 |
| **Liu QR 2023** | IV ketamine 0.25 mg/kg |  | 5 [2-8] | 2 [1-2] | 1 [0-1] |  | 163 |
|  | Control |  | 5.5 [3-8] | 2 [1-2] | 1 [1-1] |  | 163 |
| **Yang 2023** | Esketamine 2 mg kg | 4 [1-7] | 3 [1-7] | 2 [1-3] | 1 [1-2] |  | 99 |
|  | Esketamine 1 mg kg | 5 [2-7] | 5 [2-8] | 2 [1-2] | 1 [1-2] |  | 99 |
|  | Control | 7 [4-10] | 6 [3-10] | 2 [2-3] | 2 [2-2] |  | 97 |

Table 3. Summary of data that were not suitable for meta-analysism. Median (range), fregeancy (perecentage) or median [interquartile range]


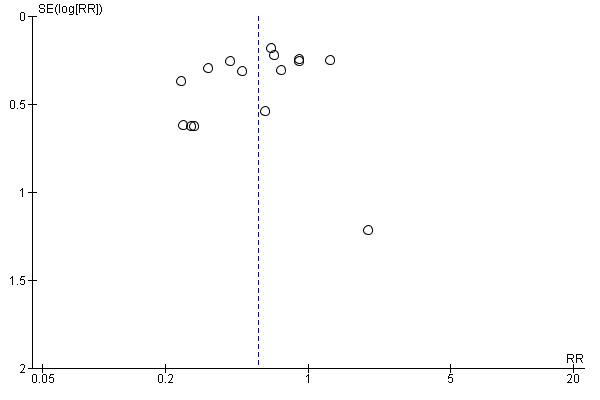
Supplementary figures

Figure 1A: funnel plot for long-term occurrence of PPD


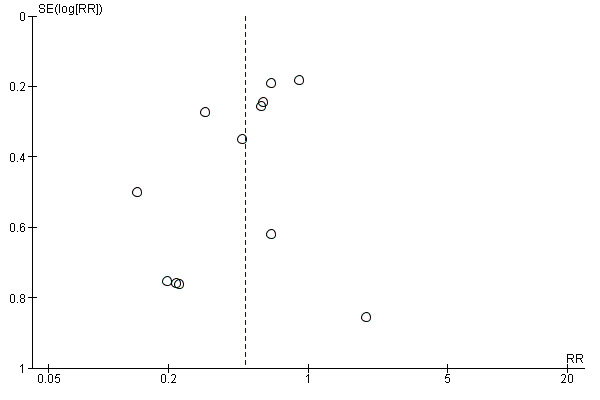


Figure 1B: funnel plot for long-term occurrence of PPD


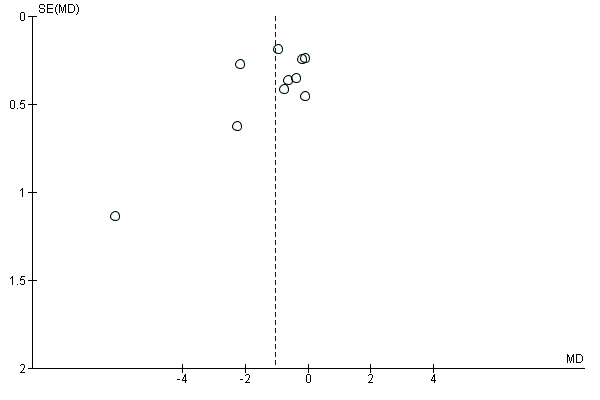


Figure 1C: funnel plot for long-term EPDS scores


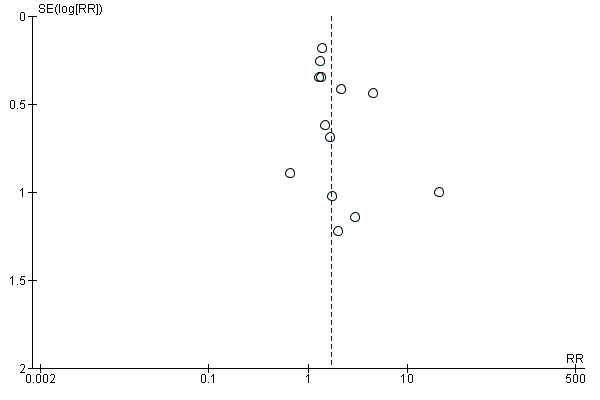


Figure 1D: funnel plot for Dizziness outcome


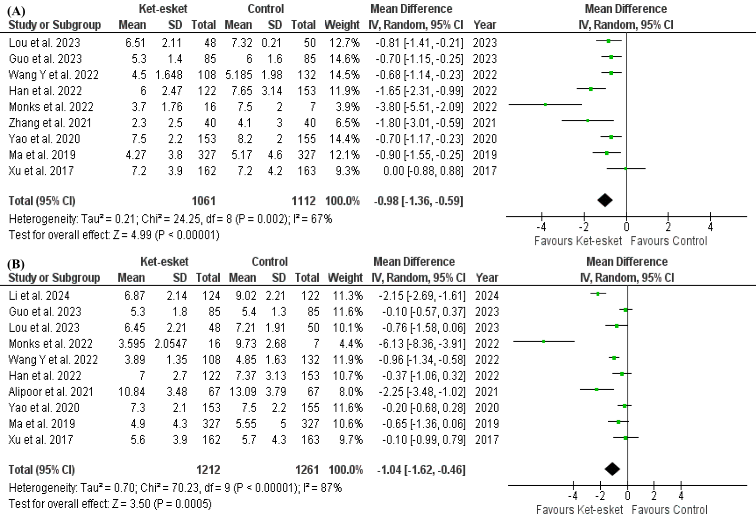


Figure 2 Forest plot A) of short-term EPDS scores, B) of long-term EPDS scores


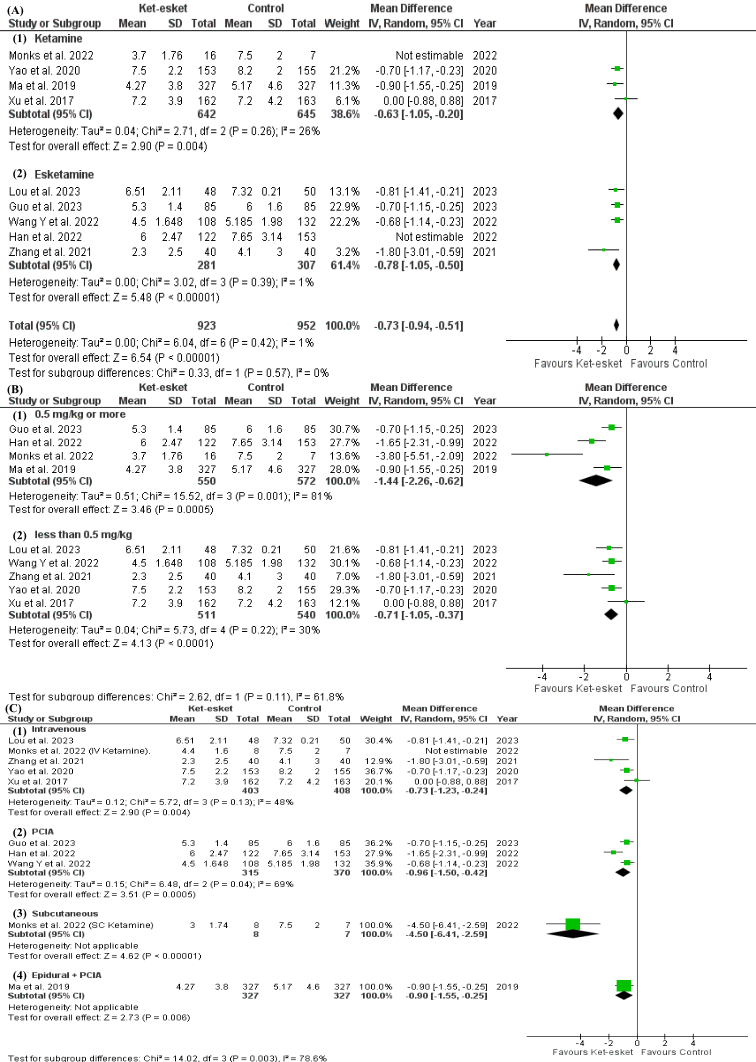


Figure 3 Forest plot A) Forest plot of subgroup analysis according to nature of drug for short-term EPDS, B) subgroup analysis according to dose of drug for short-term EPDS scores, C) of subgroup analysis according to route of drug administration for short-term EPDS scores


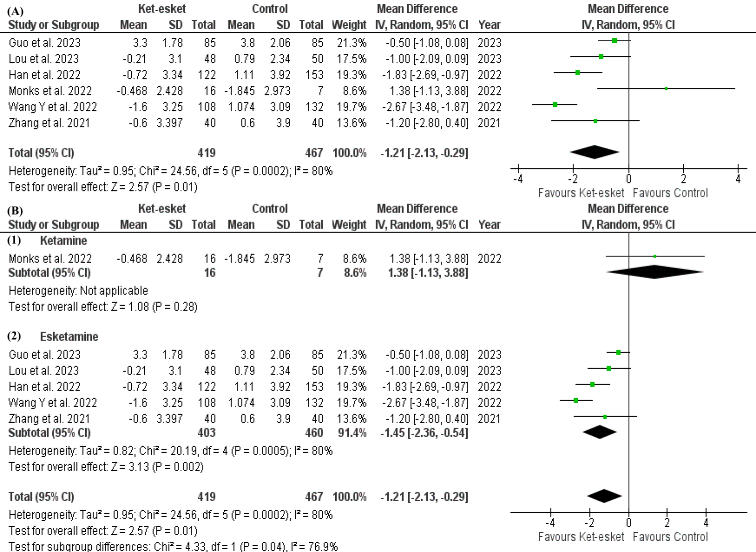


Figure 4 Forest plot A) of change in short-term EPDS scores from baseline, B) of subgroup analysis according to nature of drug for change in short-term EPDS scores from baseline


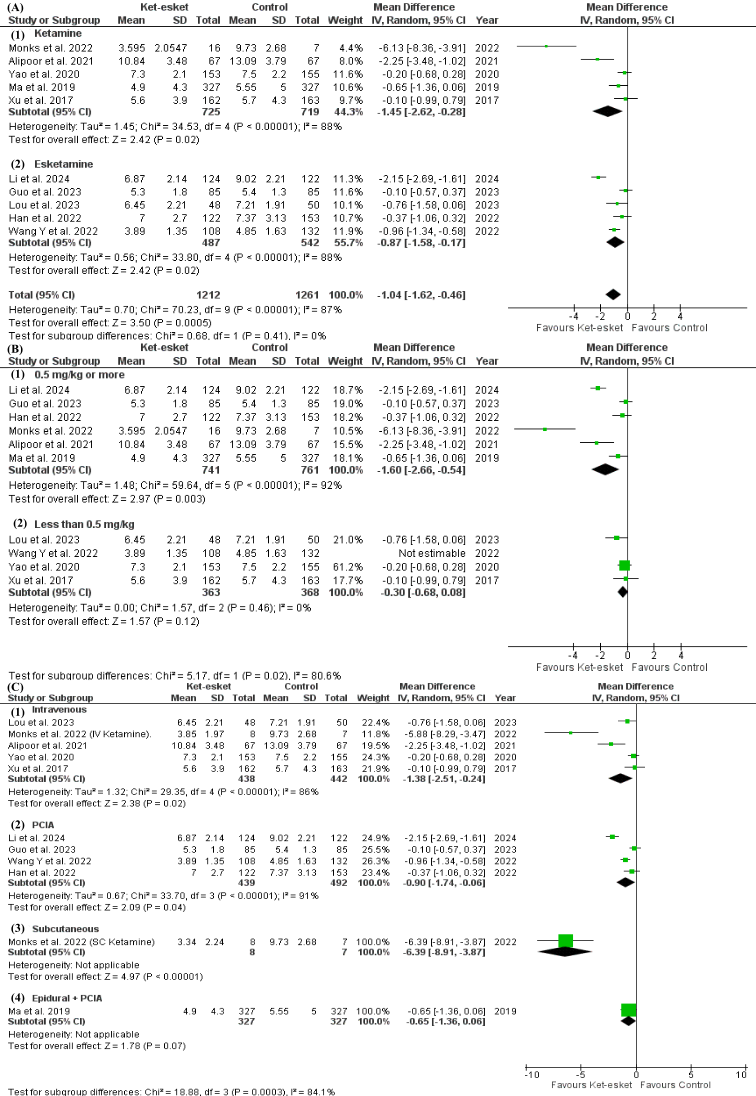


Figure 5 Forest plot A) Forest plot of subgroup analysis according to nature of drug for long-term EPDS, B) subgroup analysis according to dose of drug for long-term EPDS scores, C) of subgroup analysis according to route of drug administration for long-term EPDS scores


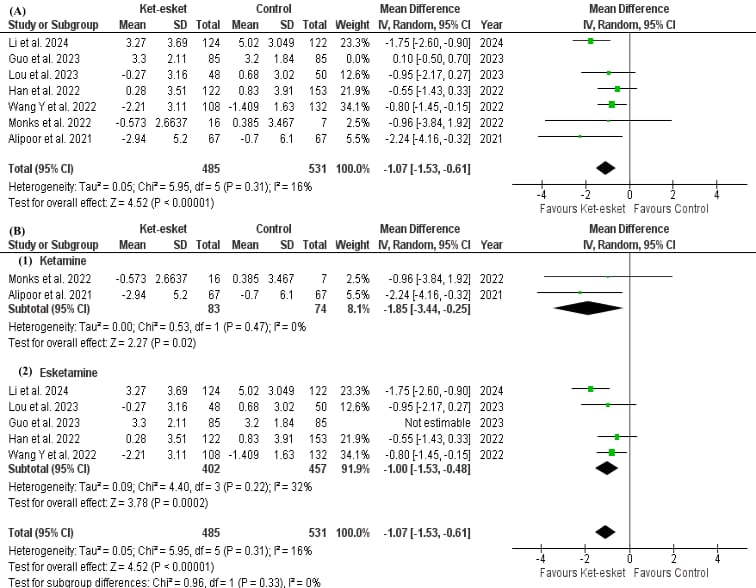


Figure 6 Forest plot A) of change in long-term EPDS scores from baseline, B) of subgroup analysis according to nature of drug for change in short-term EPDS scores from baseline


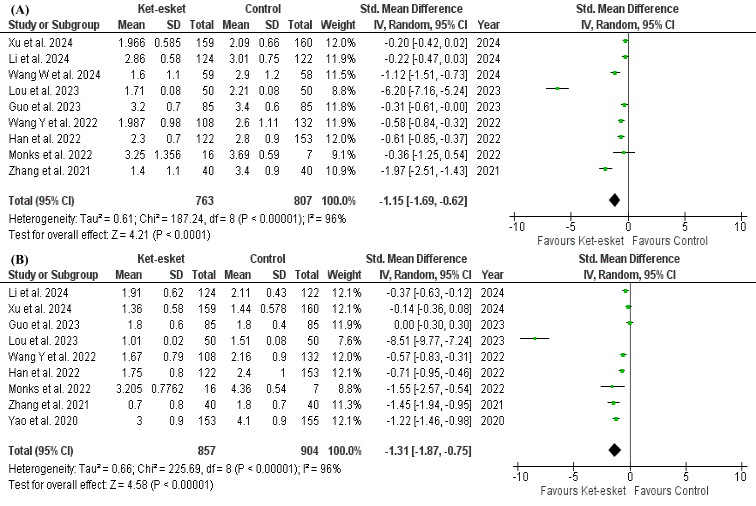


Figure 7 Forest plot A) of Day 1 pain score, B) of Day 2-3 pain score


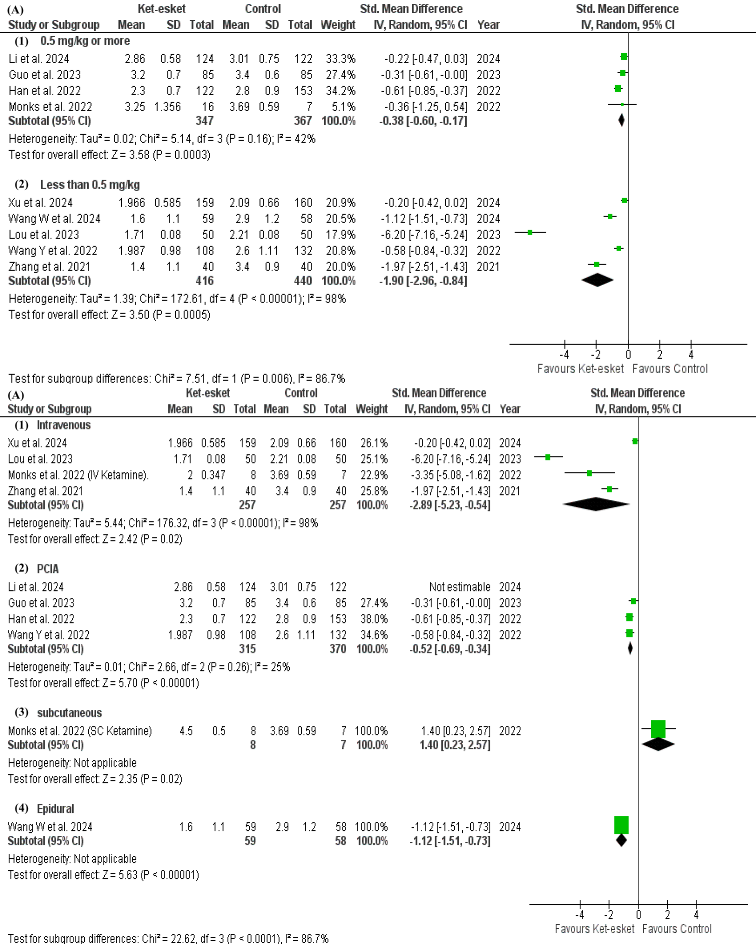


Figure 8 Forest plot A) of subgroup analysis according to dose of drug for Day 1 pain score, B) of subgroup analysis according to route of drug administration for Day 1 pain score


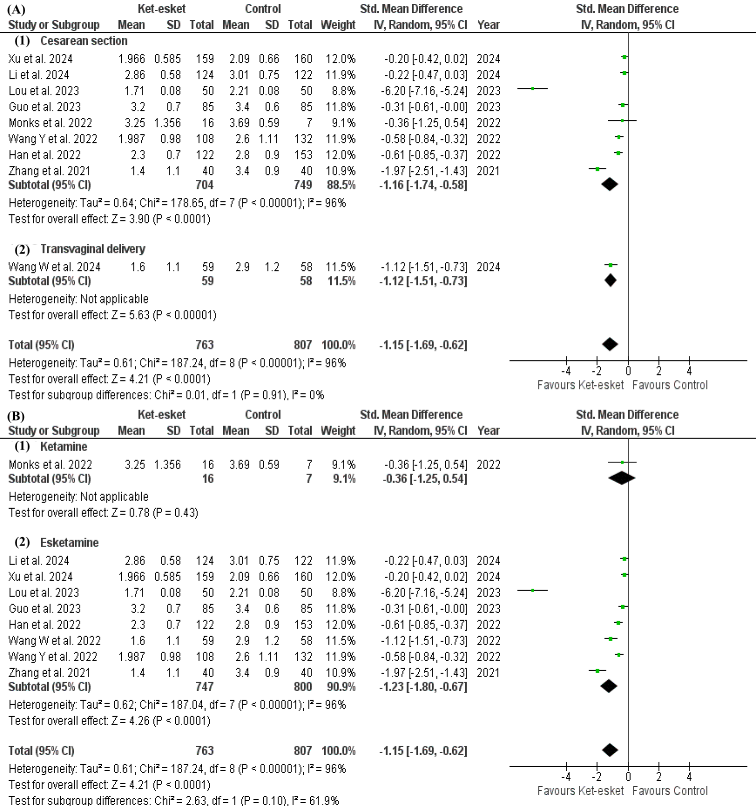


Figure 8 Forest plot A) of subgroup analysis according to mode of delivery for Day 1 pain score, B) of subgroup analysis according to nature of drug for Day 1 pain score


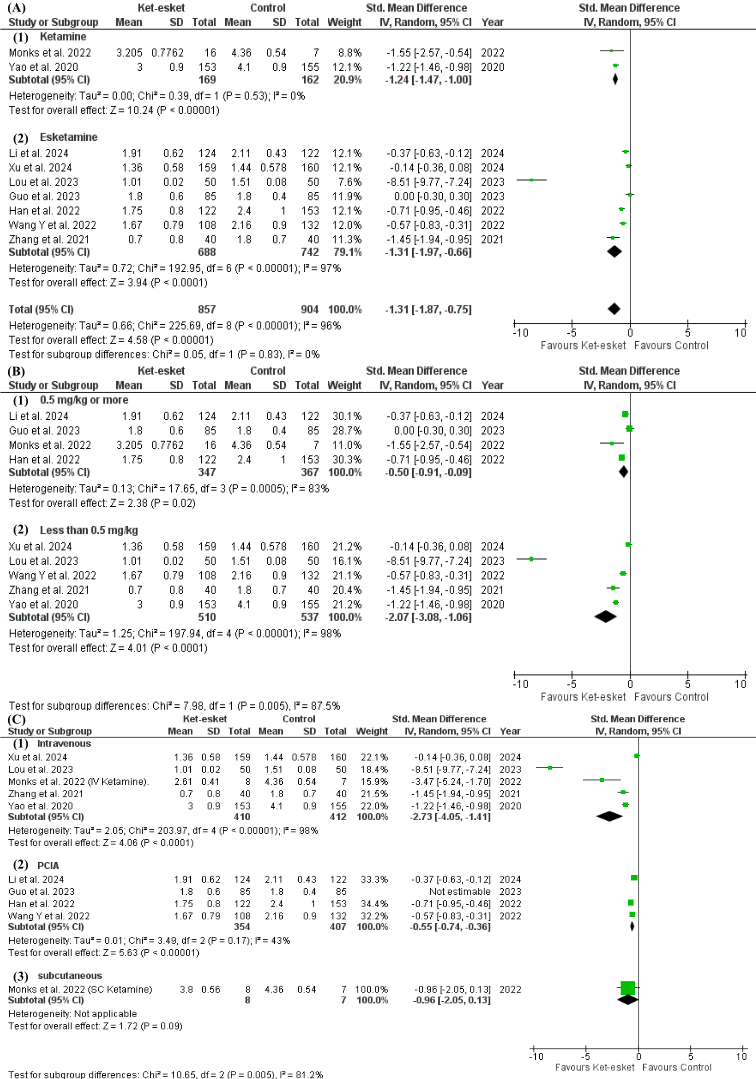


Figure 9 Forest plot A) of subgroup analysis according to nature of drug for Day 2-3 pain score, B) of subgroup analysis according to dose of drug for Day 2-3 pain score, C) of subgroup analysis according to route of drug administration for Day 2-3 pain score


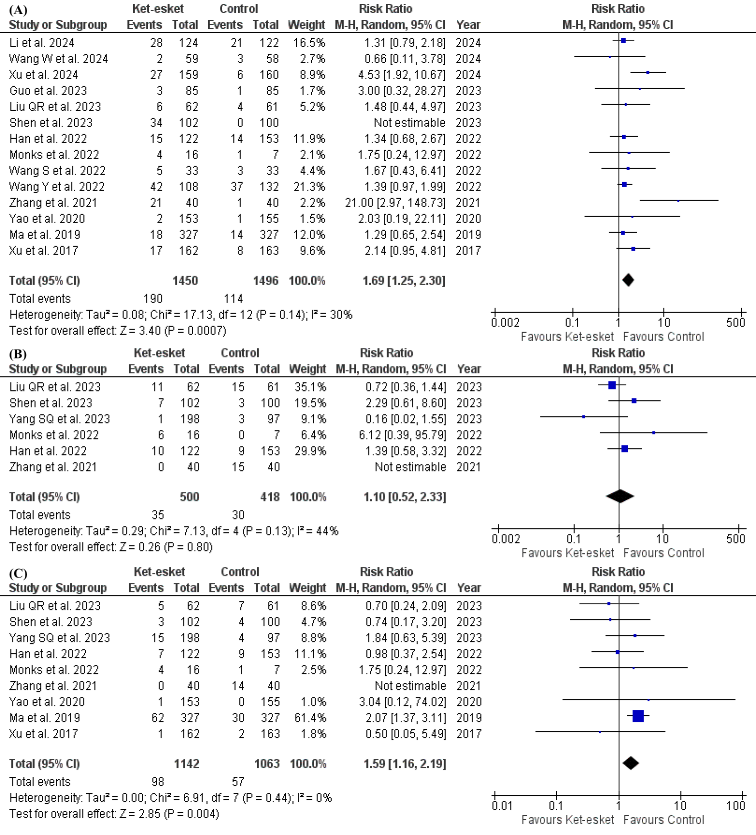


Figure 10 Forest plot A) of dizziness occurrence, B) of nausea occurrence, C) of vomiting occurrence


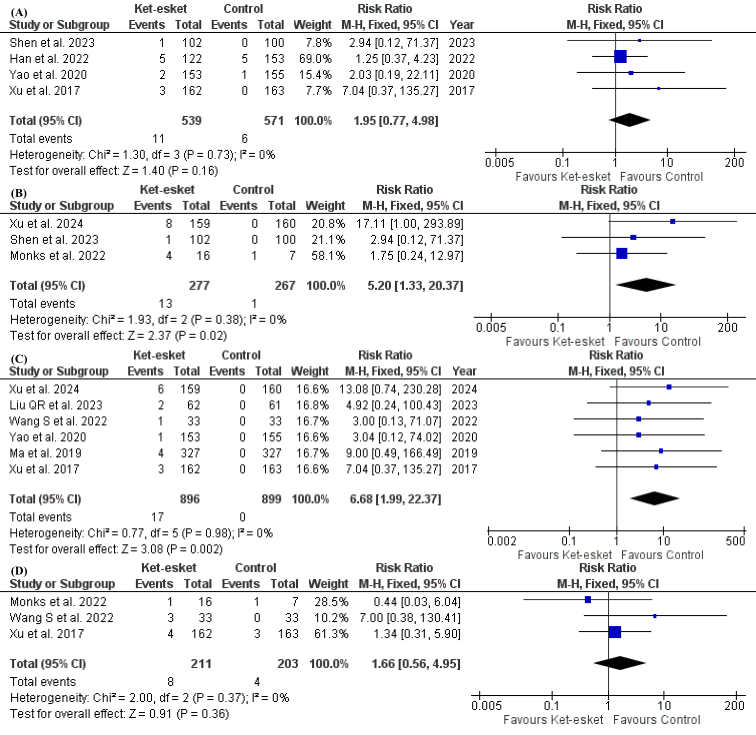


Figure 11 Forest plot A) of headache occurrence, B) of blurring of vision occurrence, C) of hallucination occurrence, D) of diplopia occurrence
